# Proteomics analysis identifies new markers associated with capillary cerebral amyloid angiopathy in Alzheimer’s disease

**DOI:** 10.1186/s40478-018-0540-2

**Published:** 2018-06-04

**Authors:** David C. Hondius, Kristel N. Eigenhuis, Tjado H. J. Morrema, Roel C. van der Schors, Pim van Nierop, Marianna Bugiani, Ka Wan Li, Jeroen J. M. Hoozemans, August B. Smit, Annemieke J. M. Rozemuller

**Affiliations:** 1 Department of Pathology, Amsterdam Neuroscience, VU University Medical Center, PO Box 7057, 1007 MB Amsterdam, The Netherlands; 2 Department of Molecular and Cellular Neurobiology, Center for Neurogenomics and Cognitive Research, Amsterdam Neuroscience, VU University Amsterdam, Amsterdam, The Netherlands

**Keywords:** Cerebral amyloid angiopathy, Amyloid beta, Alzheimer’s disease, Proteomics, Biomarker, Laser microdissection, Human brain, Post-mortem tissue

## Abstract

Alzheimer’s disease (AD) is characterized by amyloid beta (Aβ) deposits as plaques in the parenchyma and in the walls of cortical and leptomeningeal blood vessels of the brain called cerebral amyloid angiopathy (CAA). It is suggested that CAA type-1, which refers to amyloid deposition in both capillaries and larger vessels, adds to the symptomatic manifestation of AD and correlates with disease severity. Currently, CAA cannot be diagnosed pre-mortem and disease mechanisms involved in CAA are elusive. To obtain insight in the disease mechanism of CAA and to identify marker proteins specifically associated with CAA we performed a laser dissection microscopy assisted mass spectrometry analysis of post-mortem human brain tissue of (I) AD cases with only amyloid deposits in the brain parenchyma and no vascular related amyloid, (II) AD cases with severe CAA type-1 and no or low numbers of parenchymal amyloid deposits and (III) cognitively healthy controls without amyloid deposits. By contrasting the quantitative proteomics data between the three groups, 29 potential CAA-selective proteins were identified. A selection of these proteins was analysed by immunoblotting and immunohistochemistry to confirm regulation and to determine protein localization and their relation to brain pathology. In addition, specificity of these markers in relation to other small vessel diseases including prion CAA, CADASIL, CARASAL and hypertension related small vessel disease was assessed using immunohistochemistry.

Increased levels of clusterin (CLU), apolipoprotein E (APOE) and serum amyloid P-component (APCS) were observed in AD cases with CAA. In addition, we identified norrin (NDP) and collagen alpha-2(VI) (COL6A2) as highly selective markers that are clearly present in CAA yet virtually absent in relation to parenchymal amyloid plaque pathology. NDP showed the highest specificity to CAA when compared to other small vessel diseases. The specific changes in the proteome of CAA provide new insight in the pathogenesis and yields valuable selective biomarkers for the diagnosis of CAA.

**Electronic supplementary material:**

The online version of this article (10.1186/s40478-018-0540-2) contains supplementary material, which is available to authorized users.

## Introduction

Alzheimer’s disease (AD) pathology is characterized by the deposition of amyloid beta (Aβ) in the brain parenchyma as amyloid plaques and at the brain vasculature. The latter is referred to as cerebral amyloid angiopathy (CAA). Approximately 80% of AD cases have CAA pathology in varying degrees. When restricted to the larger blood vessels, including leptomeningeal vessels, cortical arteries and arterioles, this is referred to as CAA type-2. In approximately 50% of the AD cases also brain capillaries are affected, which is designated as CAA type-1 [1, 2]. Especially around the capillaries the Aβ deposits can extend into the parenchyma as perivascular Aβ also referred to as dyshoric changes [3]. In AD the observed plaque pathology and CAA type-1 capillary deposits have an inverse correlation [4].

Aβ deposition at the vessel wall in CAA correlates with an increase in the occurrence of cerebral infarction, cerebral haemorrhage and micro-bleeds. In addition, it causes a structural disruption of the vascular wall and might indirectly deteriorate the integrity of the microvascular network [5, 6]. Aβ peptide transport through the blood brain barrier (BBB) or via perivascular drainage is an important mechanism to clear the brain from Aβ. Disruption of Aβ clearance is thought to lead to increase in Aβ deposition in the walls of capillaries and blood vessels, which in turn further decreases drainage capacity resulting in further enhancement of Aβ deposition [7, 8].

CAA type-1 is clinically highly relevant, as it contributes to the symptomatic appearance of AD and, in severe form, CAA type-1 can present itself as the primary cause of rapidly progressive dementia [9, 10].

Currently, definitive diagnosis of AD and the occurrence of CAA can only be determined post-mortem. However, the presence or absence of CAA in AD patients might alter therapeutic options. In particular, a biomarker to detect CAA in patients might aid in stratification of patient groups, which is highly important when initiating, interpreting and improving outcome in clinical trials. Moreover, proteins selectively involved in CAA may function as therapeutic targets.

Proteomics analysis using mass spectrometry is a preferred method to obtain an unbiased insight into proteins involved in disease. For this, 20 cases were selected encompassing a group of AD patients with severe CAA type-1, a group with AD bearing severe plaque pathology but devoid of CAA, and a cognitively healthy control group without any pathology in the occipital lobe. Subsequently, we performed a proteomics analysis of small laser dissected occipital tissue sections containing either high plaque load, or severe CAA or no Aβ deposits. By contrasting the protein expression profiles of these subject groups we discovered proteins that are highly selective for CAA. These proteins also provide insight in specific pathogenic components of CAA, which might offer new targets for therapy.

## Material and methods

### Case selection

Post mortem brain tissue was obtained from the Netherlands Brain Bank (NBB), Netherlands Institute for Neuroscience (NIN), Amsterdam. All brain tissue was collected from donors with written informed consent for brain autopsy and the use of the material and clinical information for research purposes has been obtained by the NBB. Brain tissue was selected based on clinical and neuropathological reports. Three groups were composed. Cognitively healthy control cases lacking any pathology, AD cases with severe plaque pathology but devoid of CAA and CAA type-1 cases with severe and (nearly) pure capillary CAA pathology (Thal stages 2 and 3 for CAA) [11]. All cases are listed in Table 1. Alzheimer’s disease pathology present as Aβ deposits, neurofibrillary tangles and neuritic plaques is staged [12–14] and indicated conform the ABC criteria [15].

**Table 1:** Patient data

MS/Validation	Case	Diagnosis	M/F	Age (years)	Abeta	Tau	CERAD	PMD	APOE
MS	1	CAA type-1	F	75	A3	B3	C0^c^	6:00	44
MS	2	CAA type-1	F	96	A3	B3	C0^c^	4:20	43
MS	3	CAA type-1	M	68	A3^a^	B1	C0^c^	6:05	44
MS	4	CAA type-1	F	78	A3	NA	C0^c^	4:20	44
MS	5	CAA type-1	M	81	A3	B3	C2	6:30	44
MS	6	CAA type-1	F	95	A3	B3	C2	4:35	44
MS	7	CAA type-1	M	80	A3	B3	C0^c^	5:05	44
MS	8	AD	F	82	A3	B3	C3	6:00	42
MS	9	AD	F	72	A3	B3	C3	6:30	44
MS	10	AD	F	81	A3	B3	C3	6:00	33
MS	11	AD	F	73	A3	B3	C3	5:55	44
MS	12	AD	M	84	A3	B3	C3	8:05	NA
MS	13	AD	F	87	A3	B3	C3	5:45	43
MS	14	AD	F	72	A3	B3	C3	5:55	23
MS	15	Control	M	74	A0	B0	C0	8:05	33
MS	16	Control	F	80	A1	B1	C0	6:58	43
MS	17	Control	M	82	A0	B1	C0	5:10	23
MS	18	Control	M	78	A0	B1	C0	17:40	33
MS	19	Control	F	79	A0	B1	C0	18:13	33
MS	20	Control	F	81	A0	B1	C0	4:25	33
V	21	CAA type-1	F	94	A3	B3	C3	04:30	43
V	22	CAA type-1	M	74	A3	B3	C3	03:25	NA
V	23	CAA type-1	F	87	A3	B3	C3	08:00	44
V	24	CAA type-1	F	84	A3	B3	C2	04:45	NA
V	25	CAA type-1	M	88	A3	B3	C3	03:55	NA
V	26	CAA type-1	M	75	A3	B3	C0	03:15	NA
V	27	AD	M	64	A3	B3	C3	07:30	33
V	28	AD	F	81	A3	B3	C3	05:15	43
V	29	AD	F	90	A3	B3	C3	04:45	33
V	30	AD	M	65	A3	B3	C3	06:00	43
V	31	AD	F	73	A3	B3	C3	NA	NA
V	32	AD	F	90	A3	B3	C3	03:55	32
V	33	AD	M	88	A3	B3	C3	04:40	43
V	34	AD	M	74	A3	B3	C3	05:10	NA
V	35	Control	M	73	A0	B0	C0	24:45	33
V	36	Control	M	71	A0	B1	C0	07:40	33
V	37	Control	F	82	A0	B1	C0	07:00	33
V	38	Control	M	56	A0	B0	C0	09:15	43
V	39	Control	M	62	A0	B1	C0	07:20	33
V	40	Control	M	76	A0	B0	C0	06:45	33
V	41	Control	M	93	A0	B1	C0	05:05	33
V	42	Control	F	60	A0	B0	C0	08:10	32
V	43	Cotton wool	M	72	A3	B3	C0^c^	05:15	43
V	44	Prp-CAA	F	57	A0	B0^b^	C0	24:00	NA
V	45	CADASIL	M	73	A0	B0	C0	31:45	NA
V	46	CARASAL	F	55	A1	B1	C0	04:00	NA
V	47	Hyper tension related SVD	F	92	A1	B2	C0	07:25	NA

Alzheimer’s disease: AD, cerebral amyloid angiopathy: CAA, M: male, F: female, post mortem delay: PMD, not available/not applicable: NA, used for mass spectrometry analysis: MS, used for validation: V. (^a^Aβ only present as dysphoric CAA) (^b^Focal tau accumulation around blood vessels with prp-amyloid deposits) (^c^only dyshoric angiopathy in gallyas staining)

### Fast immunohistochemistry for LCM

Sections (10 μm) of fresh frozen occipital tissue were mounted on PEN-membrane slides (Leica), air-dried and fixed in 100% ethanol for 1 min. After air drying the tissue was wetted with sterile PBS. Anti-Aβ (clone IC16, detecting N-terminal part of Aβ [16]) was applied at a 1:100 dilution in sterile PBS (pH 7.5) and incubated for 20 min at RT. After washing 3 times for 30 s in sterile PBS, HRP labelled rabbit anti-mouse (DAKO) was applied at a 1:100 dilution in sterile PBS and incubated for 15 min at RT. Sections were briefly washed (3 × 30 s) and freshly prepared 3,3′ diaminobenzidine (DAB) solution was applied and left to incubate for 5 min to visualize antibody binding. Sections were thoroughly washed in ultra-pure H_2_O and incubated with 1% (*w*/*v*) toluidine blue in ultrapure H_2_O for 1 min as a counterstain. Sections were then washed in ultra-pure H_2_O twice for 1 min and twice in 100% ethanol for 1 min and air dried.

### Brain tissue preparation and laser capture microdissection (LCM)

Laser capture microdissection (LCM) was performed as described previously [17]. LCM was performed using a Leica AS LMD system (Leica). Cortical layers II to VI which were randomly selected from control tissue and selected based on the presence of severe Aβ pathology in the case of AD and CAA were collected in Eppendorf tubes containing 30 μl M-PER lysis buffer (Thermo Scientific) supplemented with reducing SDS sample buffer (Thermo Scientific). Between 10 and 20 tissue sections with a thickness of 10 μm were captured using LCM, yielding an equal volume each of 1.0 × 10^9^ μm^3^. Micro-dissected tissue was stored at − 80 °C until further use.

### Protein separation by electrophoresis and in-gel digestion

Micro-dissected tissue lysates were incubated at 95 °C for 5 min to denature the proteins, followed by incubation with 50 mM iodoacetamide for 30 min at RT in the dark to alkylate the cysteine residues. To reduce protein complexity, samples were size separated on a NuPAGE® 4–12% Bis-Tris acrylamide gel using MOPS SDS running buffer (Invitrogen) according to the manufacturers’ protocol.

Gels were fixed in a solution containing 50% (*v*/v) ethanol and 3% (v/v) phosphoric acid in H_2_O for 3 h at RT and stained with Colloidal Coomassie Blue (34% (v/v) methanol, 3% (v/v) phosphoric acid, 15% (*w*/*v*) ammonium Sulphate, and 0.1% (w/v) Coomassie brilliant blue G-250 (Thermo Scientific), overnight while shaking. Destaining was performed in ultra-pure water under gentle agitation for several hours to reduce background staining (Additional file 1: Figure S1 ). Each gel lane was sliced into 12 equal sized parts to reduce sample complexity during later mass spectrometry analysis and each part was cut into blocks of approximately 1 mm^3^ and collected in an Eppendorf tube. Gel fragments were destained in ultrapure water with 50 mM NH_4_HCO_3_ and 50% (*v*/v) acetonitrile overnight. Gel fragments were dehydrated using acetonitrile for 20 min and dried for 30 min using a SpeedVac. The gel parts were rehydrated in 70 μl of ultra-pure water containing 50 mM NH_4_HCO_3_ and 10 μg/ ml trypsin (sequence grade; Promega) and incubated overnight at 37 °C to facilitate digestion of the proteins. Peptides were extracted twice with a solution containing 0.1% (*v*/v) trifluoric acid and 50% (v/v) acetonitrile for 20 min. The samples were dried using a SpeedVac and stored at − 20 °C until further analysis.

### Mass spectrometry analysis

The peptides of the individual sample fractions were dissolved in 15 μL of 0.1% (*v*/v) acetic acid of which 10 μL was loaded onto a nano-liquid chromatography (nano-LC) system (Eksigent). The peptides were separated using a capillary reversed phase C18 column that had been equilibrated with 0.1% (v/v) acetic acid at a flow rate of 400 nL/min. The peptides were eluted by increasing the acetonitrile concentration linearly from 5 to 40% in 80 min and to 90% in 10 min, using the same flow rate. Eluted peptides were transferred into the LTQ/Orbitrap MS (Thermo Scientific) by Electro Spray Ionisation (ESI). The Orbitrap was operated in the range of m/z 350–2000 at a full width at half maximum resolution of 30,000 after accumulation to 500,000 in the LTQ with one microscan. The five most abundant precursor ions were selected for fragmentation by collision-induced dissociation (CID) with an isolation width of 2 Da.

### Protein inference and relative protein quantification

MaxQuant software was used for spectrum annotation, protein inference, and relative protein quantification [18]. Spectra were annotated against the Uniprot human reference proteome database (version 2016_04). Enzyme specificity was set to Trypsin/P, allowing at most two missed cleavages. Carbamido-methylation of cysteine was set as a fixed modification, and N-acetylation and methionine oxidation were set as variable modifications. Mass deviation tolerance was set to 20 ppm for monoisotopic precursor ions and 0.5 Da for MS/MS peaks. False-discovery rate cut-offs for peptide and protein identifications were set to 1% for both. The minimum peptide length was seven amino acids. Identified proteins that had the same set of peptides or a subset of peptides compared to another protein, were merged into one protein group. Peptides that were shared between different proteins were assigned to the protein with most peptide evidence (so-called ‘Razor’ peptides). Only protein groups with at least a single unique and a single Razor peptide were included. For relative protein quantification MaxQuant LFQ intensities based on at least a single shared peptide ratio were used [19].

### Statistical analysis of differential protein expression

To identify proteins that differ in abundance between the different experimental groups an ANOVA (Kruskal–Wallis test) was performed using the Perseus software platform [20], adhering to a significance cut-off of *p* ≤ 0.05. The *p* values were not corrected for multiple testing to include more proteins and provide a broad impression of the differences in the proteome.

Conditions that were set for inclusion of CAA selective proteins comprise of three approaches (A, B and C) that are visualized in Fig. 2. Approach A: T-tests (two-sided, assuming unequal variances, performed using Excel (Microsoft)) were performed contrasting the three experimental groups. When there was a significant difference (*p* < 0.05) between both the control group versus CAA, and the AD group versus CAA, a protein was labelled as CAA specific. Approach B: If the number of quantitative values in the control group was zero or one while the AD and CAA groups both had two or more quantitative values, than a *t*-test was performed between the AD and the CAA group. When the AD group had zero or one quantitative values while the control and CAA groups both had two or more quantitative values a *t*-test was performed between the CAA and control group. Approach C: In the case of zero or single quantitative values in both the control and AD groups, proteins were included based exclusively on a minimum of four quantitative values in the CAA group. Also, we included proteins with zero or single quantitative values in the CAA group and four or more values in both the AD and control groups.

ANOVA (Kruskal–Wallis test) and posthoc Dunn’s multiple comparison tests on immunoblot data and immunohistochemical data was performed using Graphpad Prism (GraphPad Software).

### Immunoblot analysis

Protein extracts were prepared by lysis of whole occipital lobe tissue in reducing SDS sample buffer using a 1:20 tissue weight to lysis buffer ratio. Proteins were denatured at 95 °C for 5 min and separated by SDS-PAGE using precast Stain Free gradient gels (Bio-Rad) and transferred (40 V overnight at 4 °C) onto a 0.45 μm PVDF membrane (Merck Millipore), which was pre-incubated in 100% methanol. The PVDF membrane was incubated in Odyssey blocking buffer for 1 h and subsequently incubated with the primary antibody overnight. After washing in Tris-buffered saline (pH 7.5) with 0.1% (*v*/v) Tween-20 (TBST) for 3 × 10 min, the membrane was incubated for 3 h with the secondary antibody. Visualization was achieved using an Odyssey imaging system using excitation wavelengths of 700 nm and 800 nm. Total protein load was visualized using a chemidoc EZ (Bio-Rad) after electro blotting (Additional file 2: Figure S2) and the protein densitometric values were then used to normalize for total protein input. Primary antibodies and dilutions are shown in Table 2. Secondary antibodies used were IRDye 800 CW Goat anti-Rabbit (LI-COR) and IRDye 680 conjugated Goat anti-Mouse (LI-COR) both were used at a 1:7.000 dilution. All anti-bodies were diluted in Odyssey blocking buffer (LI-COR). Quantification was performed using ImageJ software.

**Table 2:** Antibodies used in this study

Antibody	Source	Species	Ordernr.	Clone	Dilution (IHC)
Amyloid-beta	Kind gift of Prof. Dr. Korth, Heinrich Heine University, Düsseldorf, Germany	Mouse		IC16	1:200
APOE	Abcam	Mouse	ab1907	E6D7	1:3200
APOE	Santa Cruz Biotechnology	Mouse	sc-13521	A1.4	Used for immunoblot
NDP	Novus Biologicals	Rabbit	NBP1–84769	polyclonal	1:400
NDP	R&D systems	Mouse	MAB3014	#343711	1:800
HTRA1	R&D systems	Mouse	MAB2916	#275615	1:6400
APCS	Statens Serum Institut, SSI Antibodies	Mouse	#56585	HYB281–05	1:1600
COL6A2	Abnova	Mouse	H00001292-M01	2C5-F2	1:3200
COL6A2	Santa Cruz Biotechnology	Rabbit	SC-83607	polyclonal	1:1600

### Immunohistochemical analysis

Fresh frozen or paraffin embedded human occipital tissue was cut (5 μm). For frozen tissue the sections were placed on a SuperFrost Microscope Slide (VWR, PA, USA) and air-dried overnight at room temperature (RT). Prior to staining, the sections were fixed in 100% acetone for 10 min. For paraffin sections the paraffin was removed by washing in xylene. Next, the sections were washed in decreasing concentrations of ethanol (100%, 96% and 70% (*v*/v)). Endogenous peroxidase activity was quenched by incubating in methanol with 0.3% H_2_O_2_ for 30 min at RT. Next, antigen retrieval was performed by submerging the slides in citrate buffer (pH 6) and heating in an autoclave.

Primary antibodies were diluted in antibody diluent (VWR) and incubation was performed overnight at 4 °C. All primary antibodies and corresponding dilutions used are listed in Table 2. After incubation the sections were thoroughly washed in PBS (pH 7.4) for 30 min followed by incubation of an HRP-labelled secondary antibody, Envision (DAKO) for 30 min. Again, the sections were thoroughly washed in PBS (pH 7.4) for 30 min and then incubated with DAB to visualize antibody binding. Counterstaining of the nuclei was performed by incubation in hematoxylin for 3 min followed by extensive washing in running tab water for 5 min. Next, the slides were dehydrated by incubation in increasing concentrations of ethanol consisting of 70% (*v*/v), 96% (v/v) and 100% (v/v) ethanol. The slides were then incubated in xylene and mounted using Quick-D mounting medium. A negative control was made by omission of the primary antibody. Quantification of the staining was done using ImageJ using the threshold colour plugin.

## Results

### Selection of cases, controls and analysis of brain tissue

Three groups with a total of 20 cases were assembled based on careful neuro-pathological inspection: 1) cognitively healthy control cases (*n* = 6) without any Aβ pathology or tau pathology, 2) AD cases with severe Aβ plaque pathology but no vascular deposits (no CAA) (*n* = 7) and 3) AD cases with severe nearly pure CAA type-1 pathology and a negligible amount of plaque pathology (*n* = 7). From here, these groups will be mentioned as “control”, “AD” and “CAA”, respectively. Inclusion of these cases was done based on histochemical analysis using Congo-red and additional IHC for Aβ on the occipital frozen tissue intended for LC-MS-MS analysis.

**Fig. 1:**
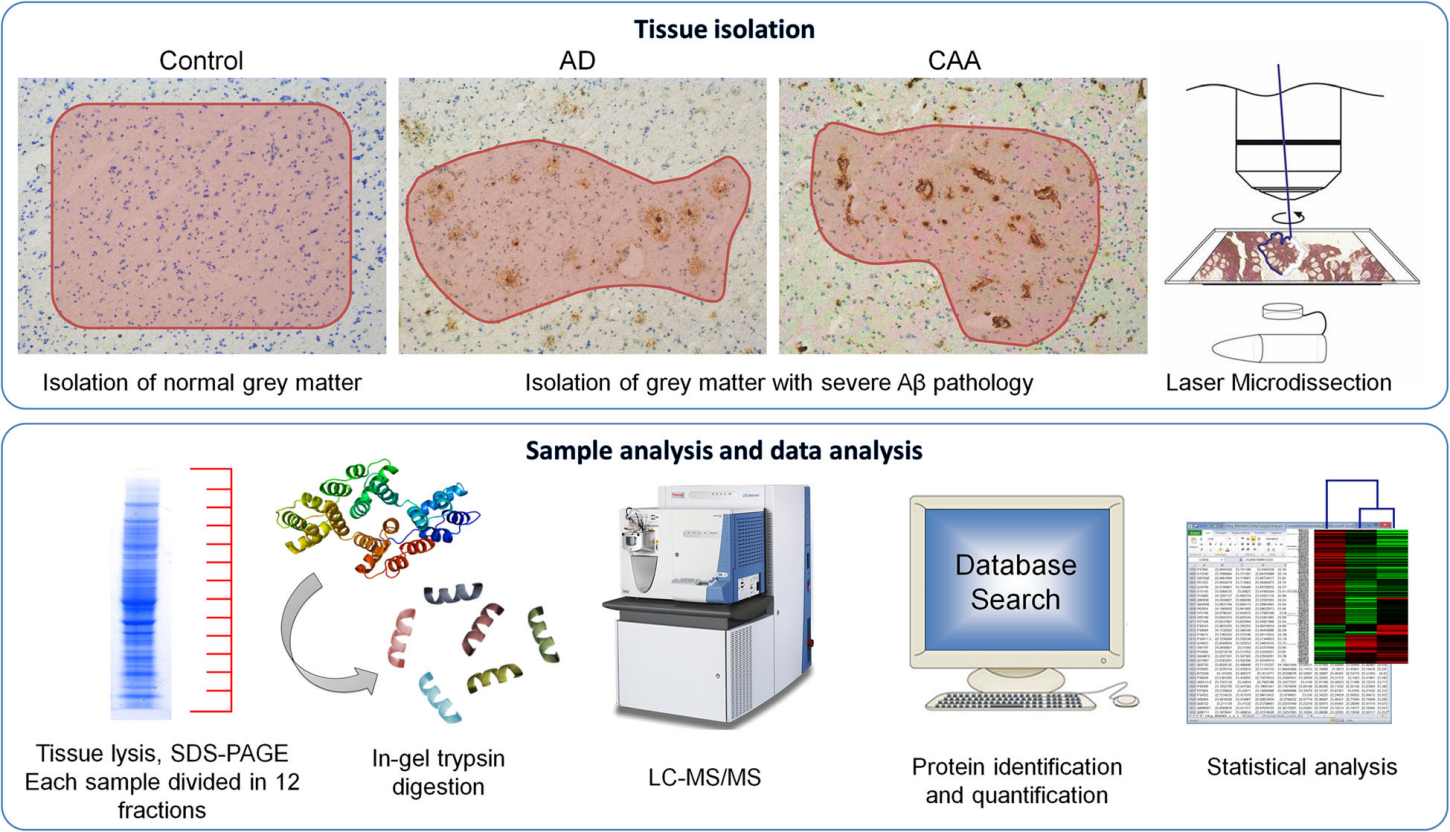
Workflow used in this study. Amyloid Beta pathology was visualized in human postmortem occipital lobe tissue. Unaffected grey matter was isolated from healthy control cases. Grey matter with high burden of Aβ pathology was isolated from the AD and CAA cases thereby isolating tissue with high plaque load or high CAA type-1 burden, respectively Tissue was lysed and the proteins were separated using SDS-PAGE and subjected to in-gel trypsin digestion. Peptides were analysed using LC-MS-MS. A database search for protein identification and protein quantification was performed using MaxQuant software. ANOVA (Kruskall Wallis) and t-tests were performed to identify significantly regulated proteins

We focussed our analysis on the occipital lobe as this region is the most frequently and severely affected by CAA pathology. Tissue sections of human occipital lobe from all selected cases were mounted on PEN-foil slides and Aβ pathology was visualized using fast immunohistochemistry. Grey matter tissue was isolated using LCM. Tissue isolation from the AD cases and CAA cases was focused on occipital lobe grey matter areas with severe Aβ pathology, i.e. high plaque load or high CAA type-1 burden, respectively. This was done to selectively enrich the input material for the proteomics analysis for these types of Aβ pathology. For control cases occipital lobe grey matter areas from the same anatomical region were selected for isolation. LCM-collected tissue samples were lysed and proteins were separated using SDS-PAGE. Each PAGE sample lane was divided into 12 fractions and subjected to in-gel trypsin digestion (Fig. 1).

### Protein quantification and global protein expression profiles

To identify and quantify proteins, liquid chromatography followed by mass spectrometry (LC-MS-MS) was performed on the 20 laser-dissected tissue samples. This allowed quantification of 2427 proteins in total and approximately 1500 proteins identified per individual case (Additional file 3: Figure S3), with a minimum of one tryptic peptide detected. All quantified proteins are listed in Additional file 4: Table S1.

To gain insight into the global similarities and differences between the three groups and the individual cases an ANOVA (Kruskall Wallis) was performed. This yielded 309 proteins that have a significant difference (*p* < 0.05) in abundance between any of the experimental groups. Using these proteins in an unsupervised clustering analysis, three different expression signatures were obtained. The protein expression signatures of the AD and CAA groups appeared largely similar, whereas both were different from the control group (Additional file 5: Figure S4A). Unsupervised clustering analysis of the individual cases using the 309 ANOVA-identified proteins, separated the controls from the disease cases (Additional file 5: Figure S4B). The CAA and AD cases were not separated on the basis of the full set of differentially expressed proteins indicating that overall their protein expression profile is largely similar. One CAA case (case #5) clustered with the control cases indicating that the protein expression profile of this sample is more similar to the control cases than to other CAA or AD cases. Visualizing the expression profile of case #5 next to the average expression profiles of the three groups confirmed the resemblance of case #5 to the control group, but also showed several proteins that are similar in expression to the AD and or CAA groups (Additional file 6: Figure S5A).

### Identification of proteins selectively altered in CAA type-1

To identify proteins that have a significantly different abundance in CAA type-1 compared to both the control and the AD group, and therefore represent unique features of CAA type-1, we performed student t-tests (two-sided, assuming unequal variances) for those proteins where at least two quantitative values per groups were available. When there was a significant difference (*p* < 0.05) between both the control group versus CAA, and the AD group versus CAA, a protein was designated as CAA-specific (Fig. 3a and Table 3a). CLU, APOE, SUCLG2, PPP2R4, KTN1, ACTG1, TNR, COL6A3 and NFASC met these criteria. In addition, levels of CLU, APOE, SUCLG2, PPP2R4 and ACTG1 were also significantly different (*p* < 0.05) when comparing the AD group with the control group.

**Table 3:** A, B and C Proteins identified as selectively altered in CAA type-1

Gene	Protein	P-val C vs CAA	P-val AD vs CAA	FDR C vs CAA	FDR AD vs CAA	FC C vs CAA	FC AD vs CAA	# detections Control	# detections AD	# detections CAA
A. Significant CAA versus control and CAA versus Alzheimer's disease										
CLU	Clusterin;Clusterin beta chain;Clusterin alpha chain	0.000	0.000	0.001	0.007	4.47	2.33	6	7	7
APOE	Apolipoprotein E	0.001	0.001	ns	ns	4.97	2.11	6	7	7
SUCLG2	Succinyl-CoA ligase [GDP-forming] subunit beta, mitochondrial	0.002	0.002	ns	ns	2.17	0.61	5	7	7
PPP2R4	Serine/threonine-protein phosphatase 2A activator	0.026	0.010	ns	ns	2.18	0.80	6	7	7
KTN1	Kinectin	0.015	0.021	ns	ns	0.34	0.40	2	2	3
ACTG1	Actin, cytoplasmic 2;Actin, cytoplasmic 2, N-terminally processed	0.000	0.035	ns	ns	0.80	0.90	6	7	7
TNR	Tenascin-R	0.007	0.017	ns	ns	0.82	0.82	6	7	7
COL6A3	Collagen alpha-3(VI) chain	0.016	0.024	ns	ns	7.79	4.95	3	5	7
NFASC	Neurofascin	0.028	0.040	ns	ns	0.86	0.87	6	7	7
B. Significant CAA versus control or Alzheimer’s disease and ≤1 detection in other group										
APP	Amyloid beta A4 protein;N-APP;Soluble APP-alpha;Soluble APP-beta;	NA	0.000	NA	ns	NA	6.65	1	7	7
UBLCP1	Ubiquitin-like domain-containing CTD phosphatase 1	NA	0.007	NA	ns	NA	1.96	1	2	4
SRI	Sorcin	NA	0.011	NA	ns	NA	0.25	1	3	5
NDP	Norrin	NA	0.020	NA	ns	NA	5.16	0	4	7
PNP	Purine nucleoside phosphorylase	NA	0.030	NA	ns	NA	1.72	0	3	3
C1orf123	UPF0587 protein C1orf123	NA	0.047	NA	ns	NA	0.71	0	6	6
DHX15	Putative pre-mRNA-splicing factor ATP-dependent RNA helicase DHX15	0.005	NA	ns	NA	0.44	NA	3	0	5
SYNPO	Synaptopodin	0.015	NA	ns	NA	0.44	NA	4	1	4
TPM1	Tropomyosin alpha-1 chain	0.021	NA	ns	NA	0.37	NA	3	1	3
CADPS2	Calcium-dependent secretion activator 2	0.042	NA	ns	NA	1.22	NA	2	1	3
SERPINA3	Alpha-1-antichymotrypsin;Alpha-1-antichymotrypsin His-Pro-less	0.042	NA	ns	NA	−0.86	NA	2	1	3
C. ≤1 detection in control and Alzheimer's disease and ≥4 in CAA OR ≤1 in CAA and ≥4 in Alzheimer’s disease and control										
HLA-DRA;HLA-DQA2	HLA class II histocompatibility antigen, DR alpha chain;HLA class II histocompatibility antigen, DQ alpha 2 chain	NA	NA	NA	NA	NA	NA	0	1	7
HTRA1	Serine protease HTRA1	NA	NA	NA	NA	NA	NA	0	1	7
APCS	Serum amyloid P-component;Serum amyloid P-component(1–203)	NA	NA	NA	NA	NA	NA	0	1	6
COL6A2	Collagen alpha-2(VI) chain	NA	NA	NA	NA	NA	NA	0	1	5
MOB2	MOB kinase activator 2	NA	NA	NA	NA	NA	NA	1	1	5
POTEI	POTE ankyrin domain family member I	NA	NA	NA	NA	NA	NA	1	0	4
KIAA1468	LisH domain and HEAT repeat-containing protein KIAA1468	NA	NA	NA	NA	NA	NA	1	0	4
TMF1	TATA element modulatory factor	NA	NA	NA	NA	NA	NA	0	1	4
SGIP1	SH3-containing GRB2-like protein 3-interacting protein 1	NA	NA	NA	NA	NA	NA	4	4	1

**Fig. 2:**
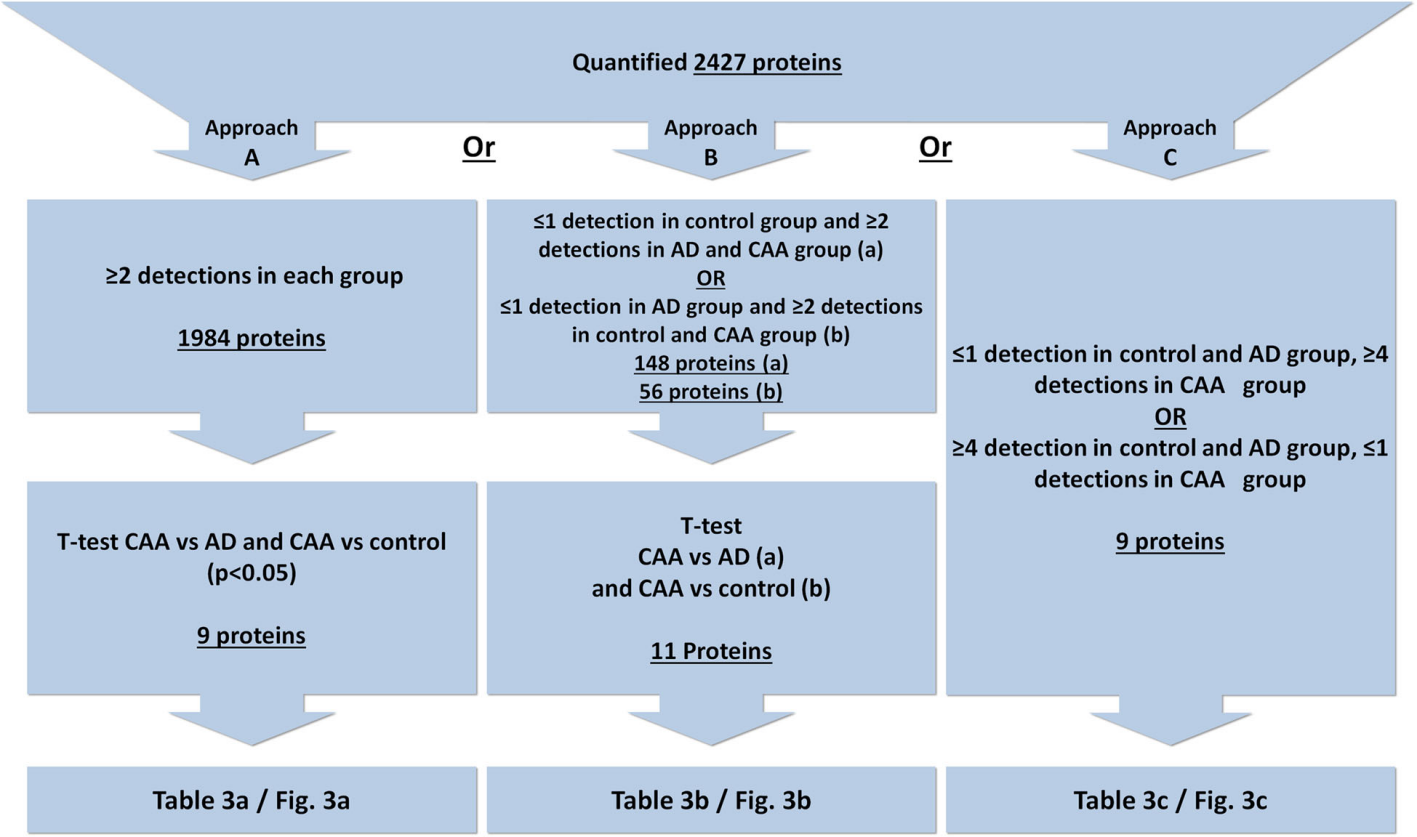
Three strategies used to select proteins that are differentially expressed in CAA type-1 compared to control and AD brains. Criteria of each of the selection strategies are specified, numbers of resulted proteins indicated, and selected proteins are listed in tables and figures as indicated

Proteins were found using the three different selection methods as described in Fig. 2

*AD* Alzheimer’s disease, *CAA* cerebral amyloid angiopathy, *FDR* false discovery rate, *NA* not applicable, *NS* not significant, *FC* fold change

After calculating the multiple testing corrected false discovery rate (FDR) only CLU was considered significant. This is likely due to the relatively low sample size of this exploratory study and the high inter-individual variance that is inevitably associated with the use of human tissue. Given the explorative nature of this study we relaxed criteria and adhered to the uncorrected *p*-values for protein inclusion.

Importantly, using label-free mass spectrometry to identify and quantify proteins, the absence of data for a number of proteins is observed. Despite great improvements in the speed and sensitivity of MS analysers missing data is almost unavoidable. When quantitative data are absent in one group while being present in the other group(s), this likely indicates differences in abundance, which might represent interesting candidate marker proteins. Therefore, absence of data in one or more patient groups required 2 additional approaches to also consider these proteins in this study. An overview of the 3 complementing strategies for protein inclusion is shown in Fig. 2 and a complete description is present in the methods section. Note that any given protein is only considered using a single approach as these approaches are mutually exclusive.

**Fig. 3:**
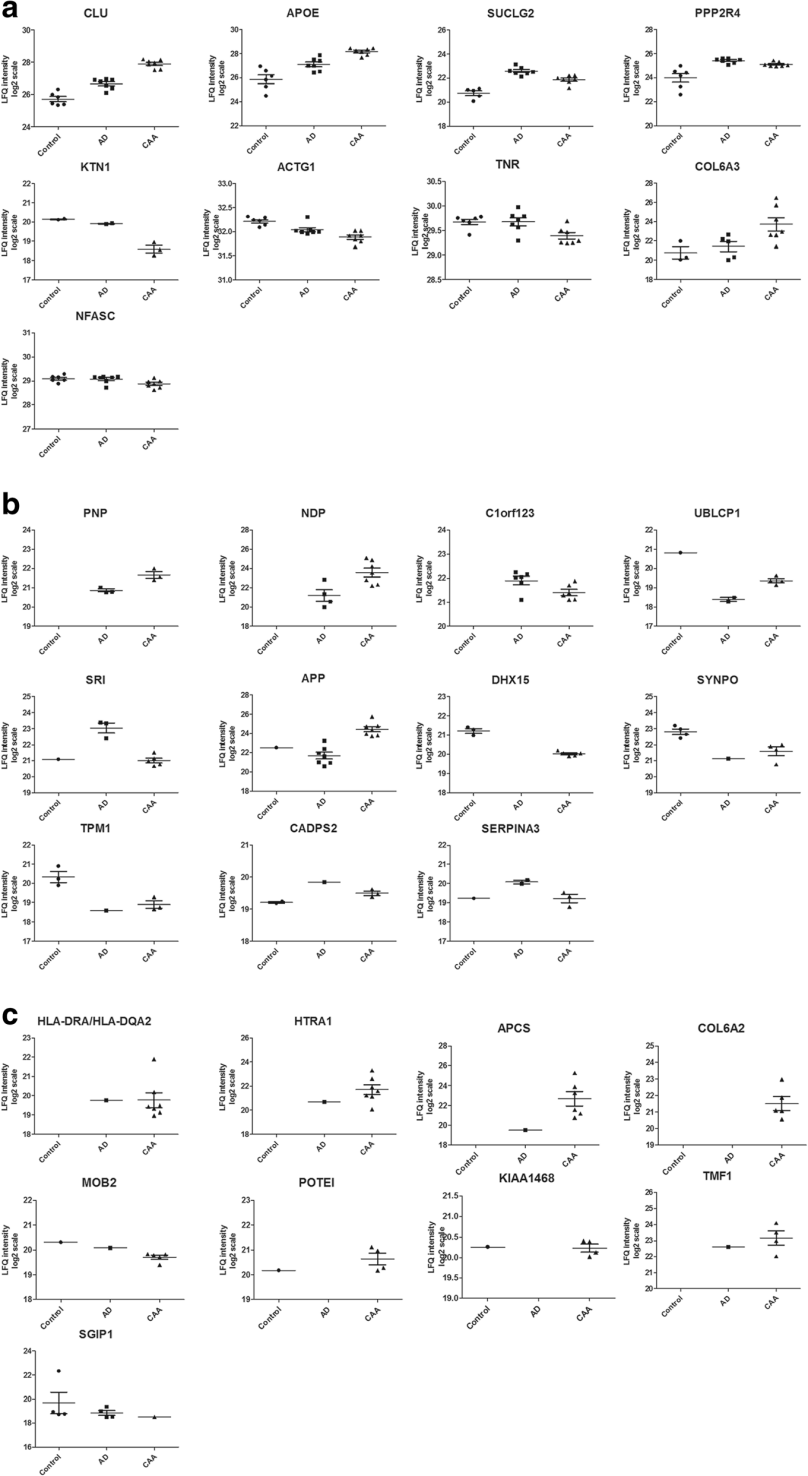
Relative abundance of proteins with altered expression in CAA type-1 compared to Alzheimer’s disease cases and controls as determined by MS. Three groups of selected proteins (panels **a**-**c**), with altered levels (MS-derived, log2 LFQ intensity values) in CAA type-1 compared to the control groups and the AD groups. Selection criteria are specified in Fig. 2. Gene names are indicated

Using approach B (Fig. 2), proteins with a significant difference were included, and APP, UBLCP1, SRI, NDP, PNP, C1orf123, DHX15, SYNPO, TPM1, CADPS2 and SERPINA3 (Fig. 3b and Table 3b) were identified as proteins selectively present in CAA type-1. Peptide data on APP indicates that quantification was based on two peptides in which the most abundantly detected peptide (LVFFAEDVGSNK) is part of Aβ.

Approach C (Fig. 2) resulted in the identification of HLA-DRA, HLA-DQA2, HTRA1, APCS, COL6A2, MOB2, POTEI, KIAA1468, TMF1 and SGIP1 (Fig. 3c and Table 3c) as CAA specific proteins.

Earlier, Case #5 was identified as having an expression profile resembling a control case. Case #5 was found positive for Alzheimer type 2 astrocytes, possibly related to high alcohol intake, and exhibited relatively low tau pathology. Otherwise, this case showed no pathological abnormalities when compared to the rest of the CAA type-1 group. However, the expression of several CAA selective markers that we identified was inspected for case #5. The levels of these markers correspond well with the other cases of the CAA group (Additional file 6: Figure S5B), indicating that these proteins are inseparably linked to the pathology of CAA type-1. In addition, although the number of cases is too small to do valid statistics, we observed no clear relation between gender and expression of the markers (Additional file 7: Figure S6).

To determine whether the above-described approaches were indeed appropriate in selecting CAA specific proteins, we performed additional immunoblotting and immunohistochemical (IHC) analysis.

### Confirmation of MS data using immunoblotting and immunohistochemical analysis

**Fig. 4:**
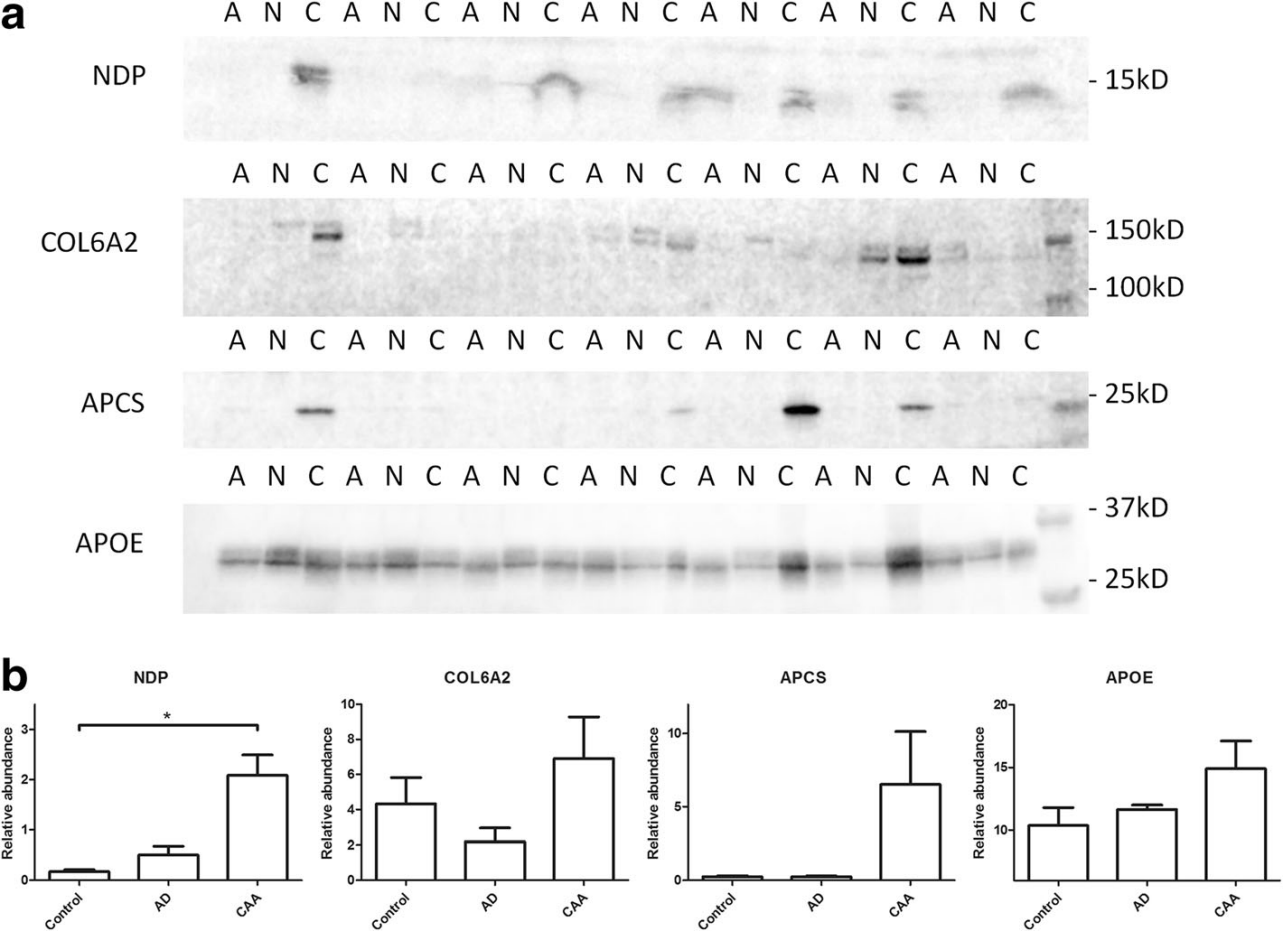
Immunoblotting analysis of proteins with altered expression in CAA type-1. **a** Immunoblotting was performed for, NDP, COL6A2, APCS and APOE on occipital lobe tissue lysates of non-demented controls (N) AD cases (A) and CAA cases (C). Immunoreactivity was observed at the correct molecular weight for each protein. **b** A significant difference (*p* < 0.05) was found in the expression of NDP in the CAA group when compared to control but not when compared to the AD group. For COL6A2, APCS and APOE significance was not reached between any of the groups using this technique. Data are expressed as mean ± SEM

Of the proteins described in Table 3 we selected APOE (approach A), NDP (approach B), HTRA1, APSC and COL6A2 (approach C), based on the fold change or specific expression in the CAA type-1 group compared to the AD and control groups, to confirm our mass spectrometry results. Immunoblotting was performed on whole tissue lysates of the same cases as used for the mass spectrometry analysis. When comparing the CAA group with the control group we found significant differences in NDP expression (Fig. 4). For APOE, APCS and COL6A2, the data showed the same trend of increased abundance in the CAA group as the proteomics data, but the differences did not reach significance. A likely explanation for this is the higher variation of expression of these proteins in the tissue used for immunoblotting, which in contrast to the mass spectrometry exploratory analysis, was not selectively enriched for pathological burden using LCM, and instead included white matter, leptomeningeal vessels and grey matter with a lower pathological burden. To unequivocally demonstrate CAA related expression, we turned to IHC analysis of these same proteins, which, in contrast to immunoblotting, allows region specific analysis similar to the LCM-LC-MS-MS analysis. For this a separate cohort was used consisting of cognitively healthy control cases (*n* = 8) without any Aβ or tau pathology, 2) AD cases with severe Aβ plaque pathology but no vascular deposits (no CAA) (*n* = 8) and 3) AD cases with severe CAA type-1 pathology (*n* = 6).

**Fig. 5:**
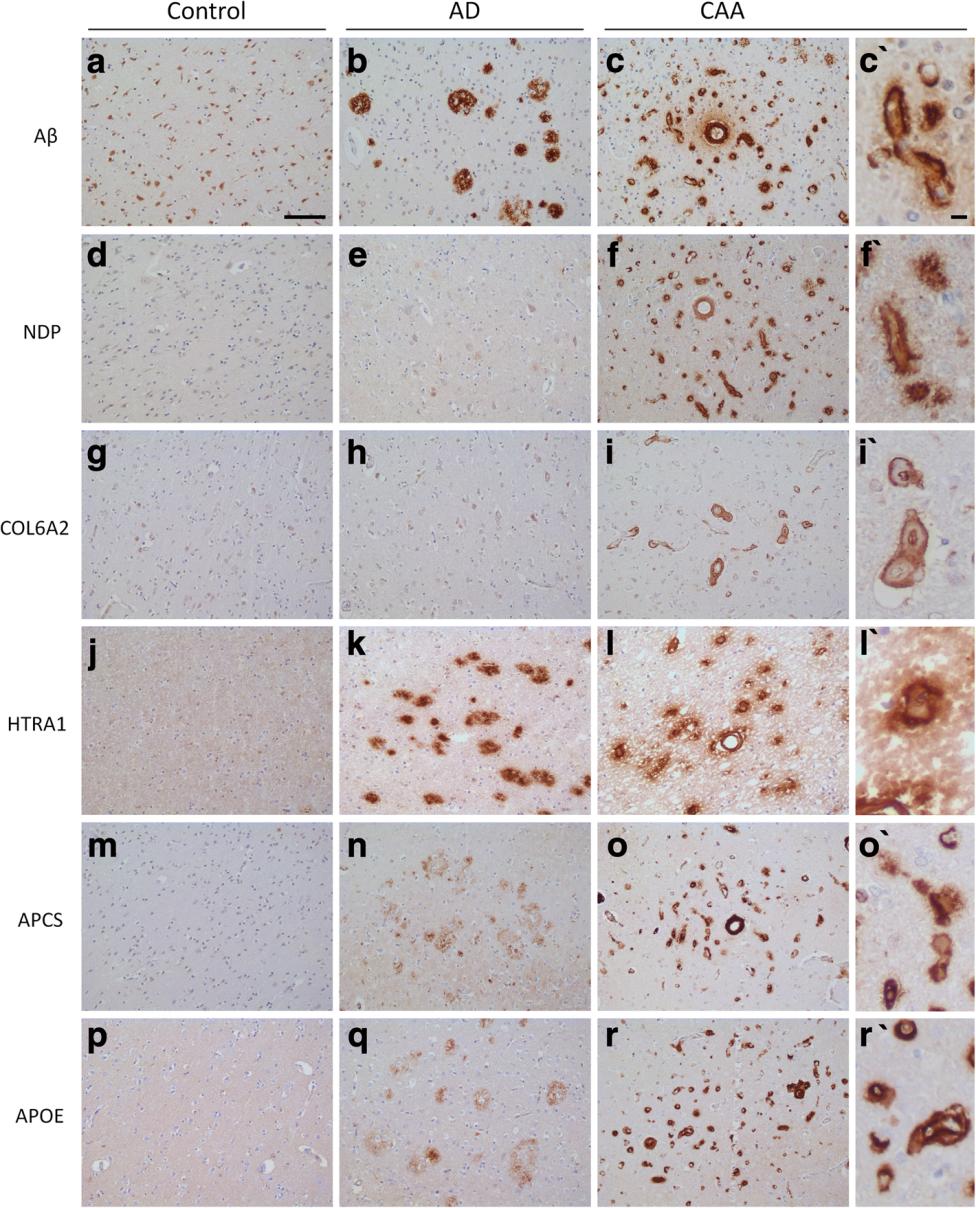
Immunohistochemical analysis of selected proteins. Representative images were taken. Aβ pathology was visualized. **a** The control case does not have any Aβ pathology. **b** Plaque pathology is confirmed in the AD case and (**c**) CAA type-1 pathology is confirmed in the CAA type-1 case. **d-f** Extensive NDP immunoreactivity is observed in the CAA type-1 cases whereas absent in both control and AD cases without CAA. **g-i** COL6A2 immunoreactivity is hardly observed in the control and AD cases, however, extensive immunoreactivity is observed in the CAA type cases and includes both capillaries and large vessels. **j-l** Immunoreactivity for HTRA1 is absent in control tissue, however, is observed both related to plaque pathology and CAA at comparable intensity. **m-o** Immunoreactivity for APCS is absent in control cases but is observed both related to plaque and CAA type-1 pathology. However, the intensity of the staining observed in the AD cases is considerably less. **p-r** Also, APOE immunoreactivity is observed related to both plaque and CAA type-1 pathology, yet its intensity in CAA type-1 is far greater. Scale bar, 100 μm in images A to R. Scale bar in image (C`) 10 μm and in all zoomed images, which are marked with a grave accent (`)

First Aβ pathology was visualized and its presence was confirmed in AD and CAA type-1 cases showing plaques and vascular Aβ pathology, respectively (Fig. 5b and c). Then, IHC analysis was performed to gain information on the localization of the selected proteins. IHC for NDP showed pronounced immunoreactivity in CAA type-1 cases that appeared associated to the vasculature. NDP immunostaining in CAA, appeared to be associated with both compact Aβ depositions as well as more diffuse staining in the parenchyma in cases that exhibit dyshoric Aβ deposits. Staining was more pronounced related to capillaries compared to larger vessels. The AD cases with plaques were nearly devoid of immunoreactivity, controls did not show any immunoreactivity for NDP (Fig. 5d-f). Different antibodies against NDP showed similar results (data not shown).

COL6A2 IHC showed some immunoreactivity in control and AD cases which was restricted to leptomeningeal vessels (Additional file 8: Figure S7) and a few large vessels in the brain tissue. In CAA type-1, immunoreactivity for COL6A2 was highly increased and includes brain capillaries and larger vessels. Immunoreactivity was mostly associated with the endothelium and/or the adventitia (Fig. 5g-i). Similar results were obtained using two different antibodies for COL6A2 (data not shown). HTRA1 IHC showed clear overlap with Aβ in both AD and CAA. Compact and diffuse staining was observed related to the vessels in the CAA cases and showed plaque pathology in the AD cases without CAA. Control cases were all negative for HTRA1 (Fig. 5j-l). IHC for APOE resulted in pronounced staining of the vasculature in CAA cases and appeared related to compact deposits as well as more diffuse dyshoric deposits. Also immunoreactivity of APOE was observed in the AD cases related to the Aβ plaques, although the staining was less intense than that related to the vascular amyloid in the CAA cases (Fig. 5p-r). APCS IHC illustrated the presence of this protein in relation with both diffuse and compact Aβ pathology in both the CAA and AD group. However, staining related to the plaque pathology was less intense than that related to the vascular Aβ pathology (Fig. 5m-o).

**Fig. 6:**
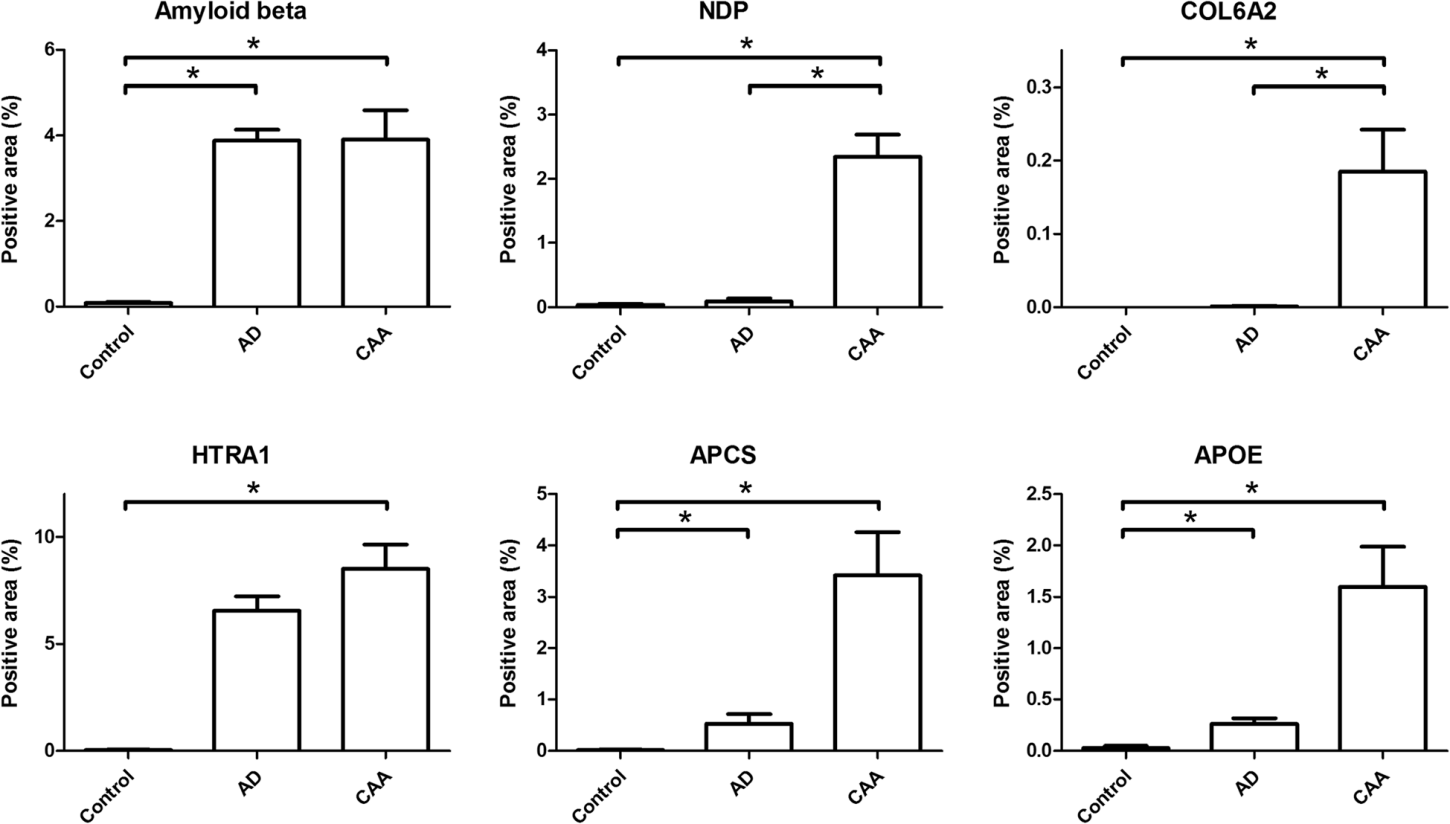
Semi-quantitative analysis of immunohistochemical data of proteins with altered expression in CAA type-1. Immunohistochemical stainings were quantified by measuring the percentage of pixels that showed positive immunoreactivity. Significance was calculated using a one-way ANOVA (Kruskal-Wallis test) and posthoc Dunn’s Multiple Comparison Test. A significant increase (*p* < 0.05) in immunoreactivity in the CAA group compared to both control and AD groups was observed for NDP and COL6A2. For APOE, APCS and HTRA1 significant differences were only found when comparing CAA with control, but not with the AD group. Data are expressed as mean ± SEM

For quantification of the IHC, images were obtained at sites that, for the AD and CAA cases, had high Aβ pathological burden in nearby sections of the same tissue block. The percentage of positively stained pixels over a total of 5 images from each case was determined. Although this method is semi-quantitative, it allowed a region-specific analysis in line with the tissue obtained by laser capture dissection that was at the basis of the original mass spectrometry analysis. Using IHC quantification we observed strongly increased immunoreactivity in CAA type-1 cases for NDP and COL6A2, and moderate increases for APCS, HTRA1 and APOE, confirming the MS results (Fig. 6).

### Specificity in relation to other small vessel diseases

**Fig. 7:**
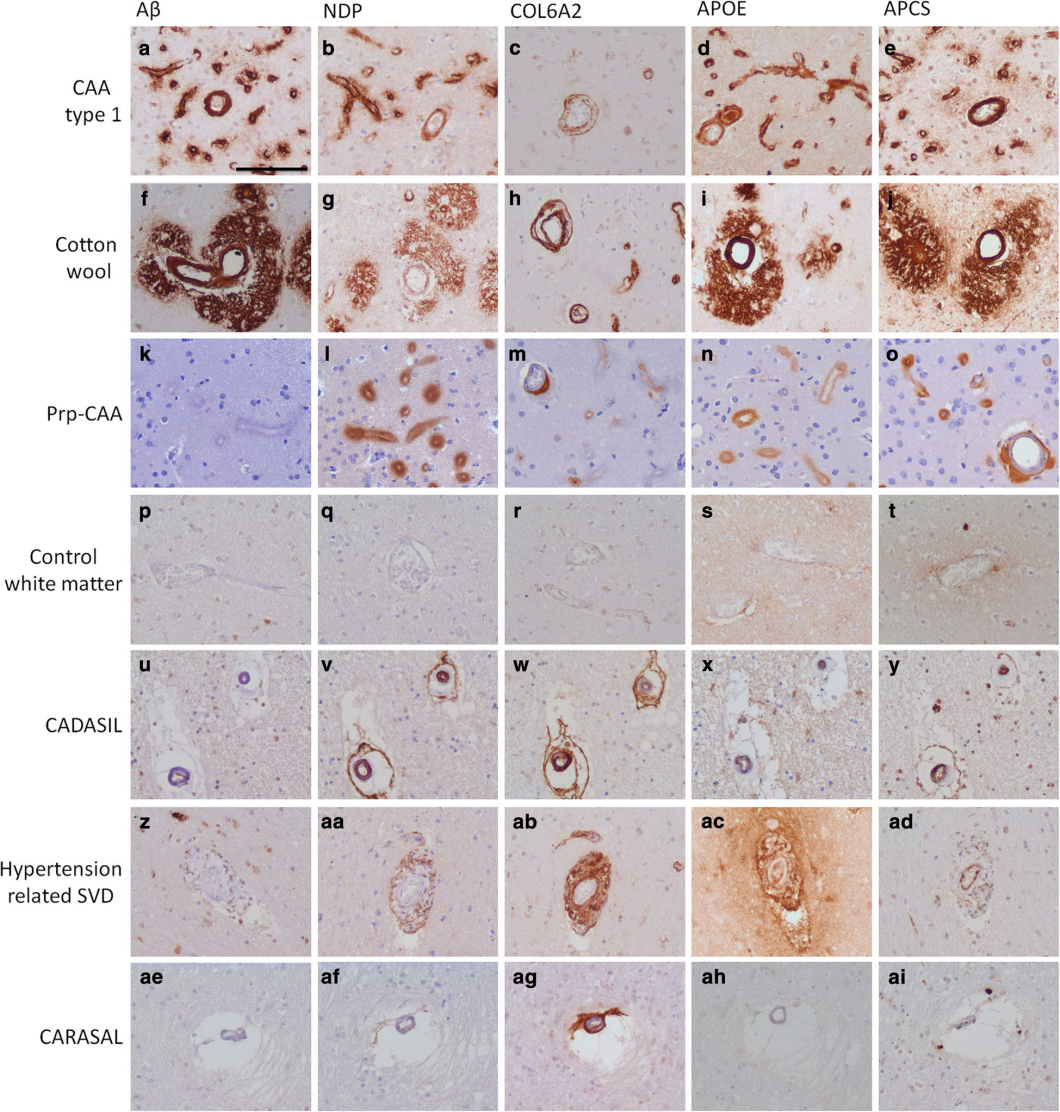
Expression of CAA type-1 markers in other small vessel diseases. IHC for Aβ, NDP, COL6A2, APCS and APOE was performed. In a CAA type-1 case immunoreactivity for all marker proteins is confirmed (**a-e**). Also immunoreactivity is seen for all markers in the cotton wool case and Aβ pathology was confirmed (**f**). For NDP, APOE and APCS immunoreactivity is also seen localizing to severe dyshoric angiopathy (**g**, **i** and **j**). COL6A2 immunoreactivity is restricted to the vessel wall (**h**). In de Prp-CAA case Aβ pathology was absent (**k**). Extensive immunoreactivity was observed for NDP, COL6A2, APOE and APCS (**l-o**). No immunoreactivity was observed for Aβ, NDP, COL6A2, APOE and APCS in the white matter of control tissue (**p-t**). In the CADASIL case no Aβ pathology was present (**u**). Mild immunoreactivity for NDP (**v**) COL62A staining was most pronounced (**w**). APOE and APCS also displayed mild immunoreactivity related to the affected vessels (**x**, **y**). In hypertension related small vessel disease no Aβ was detected (**z**) immunoreactivity of COLA6A2 was moderate (*ab*) while immunoreactivity for NDP, APOE and APCS were low but present (*aa*, *ac* and *ad*). In the CARASAL case only prominent immunoreactivity of COL6A2 was seen in affected vessels (*ag*) while NDP, APOE and APCS were absent (*af*, *ah* and *ai*). Scale bar in (*a*) indicates 100 μm

To assess the specificity of Aβ, NDP, COL6A2, APCS and APOE in relation to other small vessel diseases we performed additional IHC on cases that present various types of vascular defects, i.e., cotton wool plaque pathology, prion CAA, cerebral autosomal dominant arteriopathy with subcortical infarcts and leukoencephalopathy (CADASIL), hypertension related small vessel disease and Cathepsin A-related arteriopathy with strokes and leukoencephalopathy (CARASAL). IHC was performed on sections that exhibited the relevant pathological characteristics of each disease including an additional CAA type 1 case. Immunoreactivity for Aβ, NDP, COL6A2, APOE and APCS was confirmed in the CAA type-1 case (Fig. 7a-e). Immunoreactivity for these proteins was also assessed and confirmed in case exhibiting hereditary cerebral haemorrhage with amyloidosis Dutch type (HCHWA-D), which is a heredity form of CAA-type-1 (Additional file 9: Figure S8).

In tissue with cotton wool plaques the pathology was confirmed using IHC for Aβ displaying pathology around capillaries and larger vessels with dyshoric changes extending deep into the parenchyma (Fig. 7f). Intense NDP immunoreactivity was observed related to capillaries and the dyshoric changes and to a lesser extend directly lining the larger vessels. COL6A2 showed intense immunoreactivity lining the capillaries and larger vessels (Fig. 7g). IHC for APOE and APCS presented a highly positive, staining that appeared similar to the Aβ staining.

The PrP-CAA tissue was confirmed negative for Aβ pathology (Fig. 7k) and showed positive for Prp (data not shown). NDP immunoreactivity was highly increased and localised to the affected vessels. NDP staining was more pronounced around affected capillaries than at affected larger vessels. COL6A2 immunoreactivity was clearly present around capillaries and larger affected vessels, of which the vascular pathology was clearly observed using haematoxylin. Although co-occurring in largely the same vessels COL6A2 did not generally co-localize with the deposits, but instead localized more internally as a component of the basal membrane. APOE and APCS were also highly present and co-localized with the Prp deposits.

In the white matter no immunoreactivity was seen for Aβ or any of the marker proteins (Fig. 7p-t) in the control case. The CADASIL case showed characteristic pathology in the white matter, thickened vessel walls, in the white matter as is common with this condition. No Aβ pathology was present (Fig. 7u) Mild immunoreactivity for NDP was present and of the assessed proteins COL62A staining was most pronounced. APOE and APCS also displayed mild immunoreactivity related to the affected vessels (Fig. 7v-y).

In hypertension related small vessel disease the presence of COLA6A2 was most prominent while immunoreactivity for NDP, APOE and APCS was low but present (Fig. 7z-ad). Interestingly IHC analysis of the CARASAL cases showed only the prominent presence of COL6A2 in affected vessels while NDP, APOE and APCS were absent.

Taken our data together, from the tested panel of proteins, we recognize COL6A2 as a general small vessel disease marker. NDP, APOE and APCS are most prominent in CAA (type-1 and cotton wool) and Prp-CAA. Involvement of NDP, APOE and APCS in other small vessel diseases is varying from non (CARASAL) to mild (CADASIL). Importantly, NDP is explicitly suitable to evidently separate CAA from Aβ plaque pathology (Table 4).

**Table 4:** Scoring of relative immunoreactivity of 5 CAA-1 positive markers in different small vessel diseases and AD plaque pathology

	Aβ	NDP	COL6A2	APOE	APCS
CAA type-1	3	3	3	3	3
Cotton wool	3	3	3	3	3
Prp-CAA	0	3	3	3	3
Control white matter	0	0	0	0	0
CADASIL	0	2	2	1	1
Hypertension	0	1	2	1	1
CARASAL	0	0	2	0	0
AD plaques	3	0	0	2	2

0: no immunoreactivity, 1: mild immunoreactivity, 2: moderate immunoreactivity, 3: extensive immunoreactivity

## Discussion

One of the most prevalent cerebro-vascular diseases in the elderly is sporadic CAA, characterized by vascular deposition of amyloid-beta protein. CAA can occur as an isolated disease or as part of the pathology in AD. Several studies have indicated CAA as an important cause of cognitive decline [10, 21, 22]. Currently, there is no treatment for CAA and its presence cannot be diagnosed pre-mortem. Therefore insight in the pathogenic mechanisms and the need for biomarkers are urgent. We performed an exploratory laser dissection-assisted LC-MS-MS analysis of AD brain tissue exhibiting severe CAA type-1 pathology, AD brain tissue without apparent involvement of CAA, and control brain tissue without AD related pathology. We show that the proteome of CAA type-1 is different from that of parenchymal plaque pathology in AD, which led to the identification of proteins selectively associated with CAA.

Next to identification of new CAA selective proteins, this study also confirmed the presence proteins already known to be involved in CAA pathology, e.g., CLU, APOE and APCS [23, 24]. Interestingly, CLU was detected in all samples included in the study and its abundance was sufficient to completely separate the CAA group from both the control group and the AD group. Moreover, CLU, APOE and APCS were markedly increased in AD compared to controls and APOE and APCS showed moderate immunoreactivity related to plaque pathology, in accordance with previous findings [24, 25]. Interestingly, increased levels of CLU have been reported in the plasma of CAA patients diagnosed according the modified Boston criteria [26].

Two recent proteomics studies focussed on CAA analysing leptomeningeal vessels [27] and leptomeningeal vessel combined with neocortical arterioles [28]. Some similarities were found with these studies, such as the increase in CLU and APOE. As expected also several differences exist as our analysis focussed on grey matter of CAA type-1 using micro dissected tissue that is enriched for areas with very high capillary associated Aβ pathology. These differences might indicate that other mechanisms are involved in the pathogenesis in CAA related to capillaries compared to larger vessels, e.g., the findings of NDP and COL6A2. In contrast to these previous studies we compared CAA cases with both control and AD cases with plaque pathology and without CAA. There are many similarities in the response to CAA and AD and these proteins and processes can be cancelled out against each other, allowing CAA selective proteins to become apparent.

Importantly, we identified potential new key players in the development of CAA. NDP is found highly upregulated in CAA type-1, cotton wool Aβ pathology and Prp-CAA, and localizes around the affected vasculature. NDP immunoreactivity was only mildly increased in CADASIL and hypertension related small vessel disease. In addition, these diseases affect different anatomical regions, clearly identifiable with imaging studies, and present a distinct clinical picture, leaving NDP a promising biomarker for CAA.

NDP is a small, secreted protein with a molecular weight of approximately 15 kD. It has important function in the formation of the brain vasculature during development and in maintenance of a proper functioning BBB [29]. In the adult brain the NDP gene is primarily expressed by astrocytes [30]. NDP activates the canonical Wnt/β-catenin signalling pathway via the frizzled (Fzd)4/low-density lipoprotein receptor-related protein (Lrp)5/6 receptor complex [31].

In neural progenitor cells (NPCs) derived from FAD mutant PSEN1 subjects it was found that NDP mRNA is upregulated, but no increase in mRNA was found in AD human temporal lobe [32]. In the retina NDP was found to promote regrowth of capillaries and formation of intra-retinal vessels after oxygen-induced retinal damage [33]. Mice overexpressing NDP, had significantly less vascular loss following oxygen exposure. Mutations in the NDP gene result in Norrie disease, which is primarily an eye disease that leads to blindness. Interestingly, 30–50% of these patients display developmental delay, intellectual disability, behavioural abnormalities, or psychotic-like features [34, 35]. In addition, NDP has been shown to protect neurons against excitotoxicity induced by NMDA [36]. NDP seems to have protective properties for both endothelial cells and neurons, but whether NDP upregulation is beneficial in the context of CAA pathology is unknown.

Our proteomics data show COL6A2 expression in CAA cases, which is supported by a strong increase in COL6A2 immunoreactivity in the affected brain parenchyma. In addition to COL6A2 we also found COL6A3 highly increased in CAA type-1. COL6A2 immunoblotting did not show a significant difference between the experimental groups. This can be explained by the inclusion of leptomeningeal vessels, that express high levels of COL6A2 in all cases in varying amounts in the tissue lysates used for immunoblotting, as shown using IHC. COL6A2 was also found increased in the other small vessel diseases that were included for IHC analysis. This indicates that COL6A2 might be used as valuable indicator of vascular pathology in general, but not specific for CAA type pathology. COL6A2 is a non-fibril collagen and COL6 isoforms are present in various tissues including the vasculature [37]. COL6A2 encodes one of the three alpha chains of type VI collagen, which is found in most connective tissues. Type VI collagen anchors endothelial basement membranes by interacting with type IV collagen [38]. In the brain, collagen VI was shown neuroprotective and its expression increased in animal models of AD [39].

HTRA1 is a trypsin-like serine protease which was detected in all CAA samples, with a single value in the AD group and zero quantitative values in the control group. Using immunohistochemistry we found a significant difference with the control groups but not with the AD group as HTRA1 marks normal plaque pathology as well. HTRA1 is relevant in neurodegeneration as this protease is involved in the degradation of APP and Aβ [40]. In addition, HTRA1 was found to degrade APOE4 more efficiently than APOE3 and the presence of APOE4 reduces digestion of MAPT by HTRA1 [41]. Moreover, mutations in HTRA1 are the cause of the hereditary small vessel disease CARASIL (cerebral autosomal recessive arteriopathy with subcortical infarcts and leukoencephalopathy) [42, 43].

As part of our LC-MS-MS exploratory study we identified other proteins that are potentially interesting for additional research in relation to CAA, but were not specifically followed up in this study. For instance, we observed significant high levels of HLA-DR/HLA-DQ in CAA type-1. This protein is associated with inflammation and high numbers of activated microglia [4].

PNP for which a single nucleotide polymorphism was found to be associated with faster progression of AD [44]. Its relation to CAA is yet unknown.

SUCLG2 which is involved in clearance of Aβ1–42 [45] was found increased in CAA compared to control and even higher levels were observed in the AD cases. APP was identified with increased levels in CAA. Peptide data indicates that the most abundantly detected peptide is a part of Aβ, however this analysis cannot discriminate between Aβ or APP.

The proteins in this study, that show selective association with CAA type-1 pathology might serve as potential CAA type-1 biomarkers in patients. As larger vessels are also positive for the markers that were assessed using IHC, these markers might also be relevant in CAA type-2, although in the case of NDP the intensity of immunoreactivity is less in larger vessels compared to affected capillaries in CAA.

CAA selective markers might be used for pathological assessment of the severity of CAA. The association of Aβ with the vasculature, and in particular capillaries, is not always obvious in thin microscopic sections. Also, the use of these proteins as potential diagnostic markers should be explored.

The need for a biomarker for CAA is urgent, in part for (early) diagnosis of CAA, but also for stratification of patients involved in clinical trials for AD. For instance, anti-amyloid immunotherapies in development may warrant separation of AD patients with or without CAA because of expected side effects associated with CAA, including vasogenic oedema and cerebral microhemorrhages [46, 47]. In addition, these markers would help to improve the assessment of the safety of anticoagulation therapy in patients with CAA as they increase the risk of intracerebral haemorrhage [48].

## Conclusion

In conclusion, we present a set of marker proteins containing known and new markers representing valuable tools for both clinical and neuropathological diagnosis which can contribute to studies investigating the role of CAA in AD pathology. In addition to their use as biomarkers, the newly found proteins might be further investigated to increase our understanding of etiology and disease mechanism related to CAA, and ultimately may be used as therapeutic targets.

## Additional files

Additional file 1: **Figure S1.** Coomassie blue staining of the SDS PAGE gels containing the microdissected tissue lysates. (TIF 478 kb)

Additional file 2: **Figure S2.** Total protein fluorescent signal from blots used for immunoblot analysis. Total protein load was visualized using a chemidoc EZ (Bio-Rad) after electroblotting and used to obtain densitometric values which were then used to normalize for total protein input. (TIF 553 kb)

Additional file 3: **Figure S3.** Number of proteins detected per individual case. Proteins were quantified based on a minimum of one peptide and adhering to an FDR of < 0.01. (TIF 19662 kb)

Additional file 4: **Table S1.** Complete dataset, containing log2 transformed quantitative values (LFQ values) of all quantified proteins per individual case. (XLSX 665 kb)

Additional file 5: **Figure S4.** Clustering analysis of experimental groups and individual cases. Clustering analysis and heat maps of the different experimental groups (A) and individual cases (B) based on proteins with a significant difference (ANOVA, *p* < 0.05) in expression between any of the groups. (TIF 709 kb)

Additional file 6: **Figure S5.** Protein expression of CAA case #5 relative to the experimental groups and individual cases. (A) On the left the expression profile of case #5 compared to the average expression profile of the control group (2nd row), AD group (3rd row) and the CAA group (4th row). Green, expression below the overall mean; red, above the overall mean. The expression profile of case #5 is largely similar to that of the control groups but some proteins show a similar expression as in the AD and/or CAA groups. (B) Expression values (LFQ values) of several CAA specific proteins identified in this study with case #5 indicated as empty triangle pointing down. Case #5 does not differ from the CAA group in these markers. (TIF 1835 kb)

Additional file 7: **Figure S6.** Protein expression of males versus females. Quantitative data on several CAA selective data was plotted with males represented as triangles and females as dots. No clear relationship between gender and protein abundance was observed. (TIF 24739 kb)

Additional file 8: **Figure S7.** Immunoreactivity for COL6A2 is equally present in leptomeningeal vessels in control, AD and CAA tissue. (TIF 3794 kb)

Additional file 9: **Figure S8.** Immunohistochemistry of Amyloid-beta, NDP, COL6A2, APOE and APCS on multiple brain regions of a HCHWA-D CAA type-1 case. Brain tissue of a case exhibiting a hereditary form of CAA type-1 was analyzed by immunohistochemistry of Amyloid-beta, NDP, COL6A2, APOE and APCS. Aβ pathology was confirmed and immunoreactivity associated with CAA type-1 pathology was found present for all markers. Scale bar in upper left picture represents 100 μm. (TIF 34988 kb)
