# Supplementary figures and images for: Proteomics analysis identifies new markers associated with capillary cerebral amyloid angiopathy in Alzheimer’s disease
Source: Acta Neuropathol Commun. 2018 Jun 4;6:46. doi: 10.1186/s40478-018-0540-2 (PMC5985582; doi:10.1186/s40478-018-0540-2)

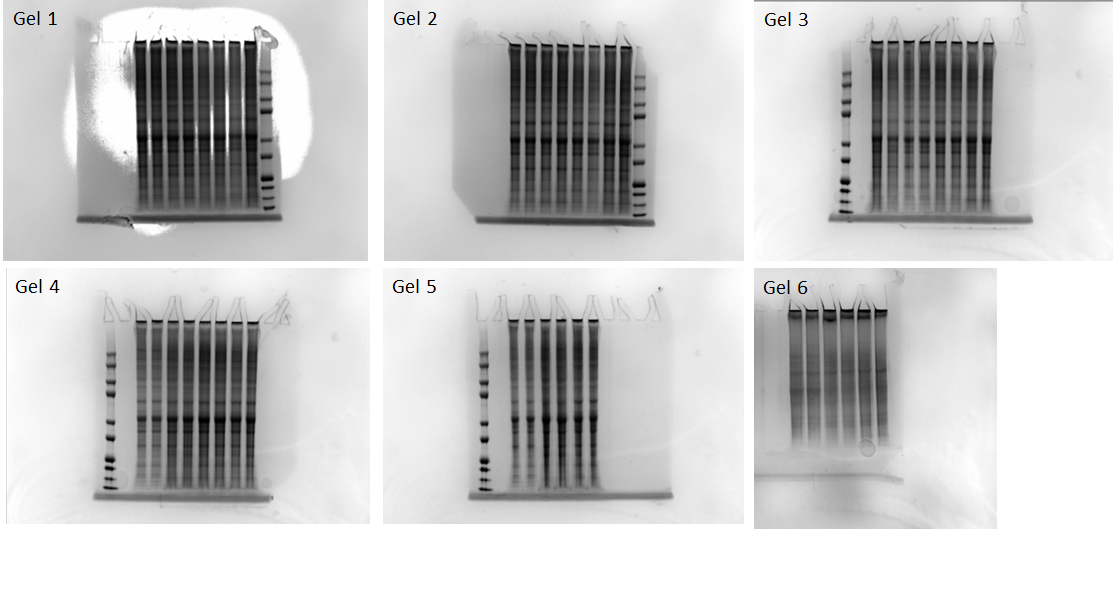

Supplement: Supplementary file 1 — Figure S1. Coomassie blue staining of the SDS PAGE gels containing the microdissected tissue lysates. (TIF 478 kb) [file 40478_2018_540_MOESM1_ESM.tif]

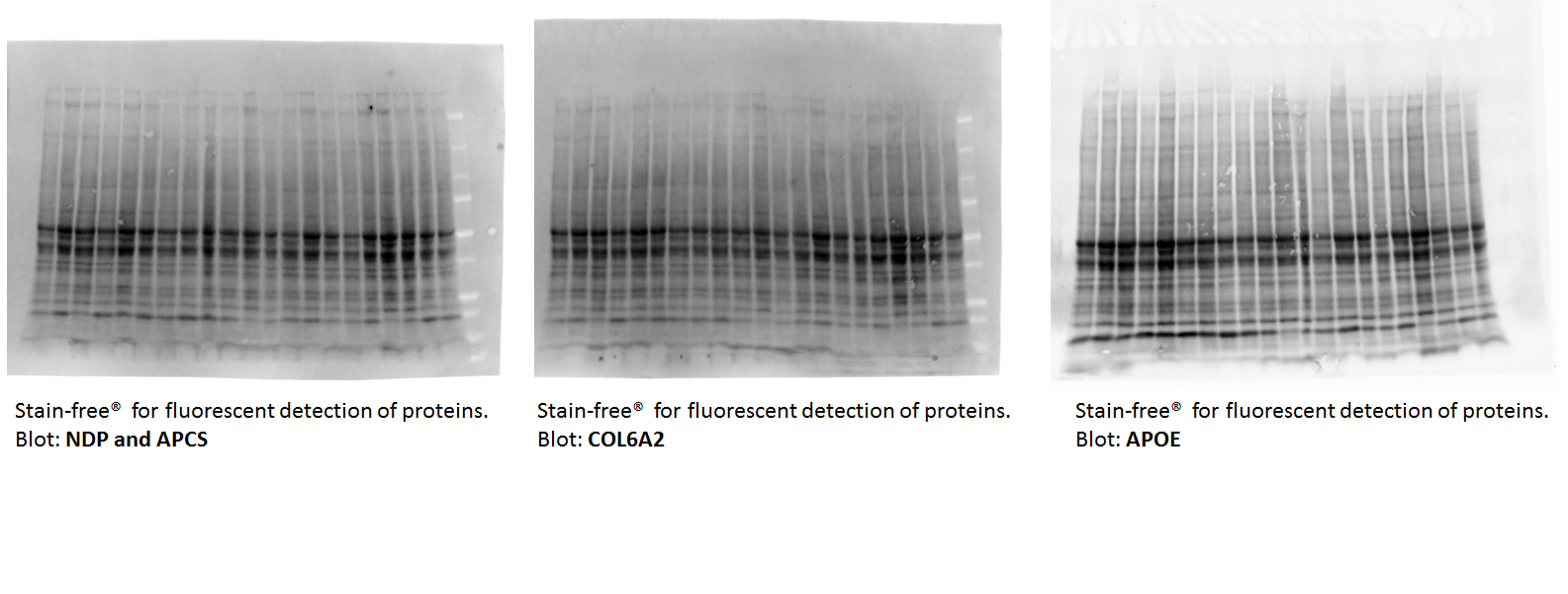

Supplement: Supplementary file 2 — Figure S2. Total protein fluorescent signal from blots used for immunoblot analysis. Total protein load was visualized using a chemidoc EZ (Bio-Rad) after electroblotting and used to obtain densitometric values which were then used to normalize for total protein input. (TIF 553 kb) [file 40478_2018_540_MOESM2_ESM.tif]

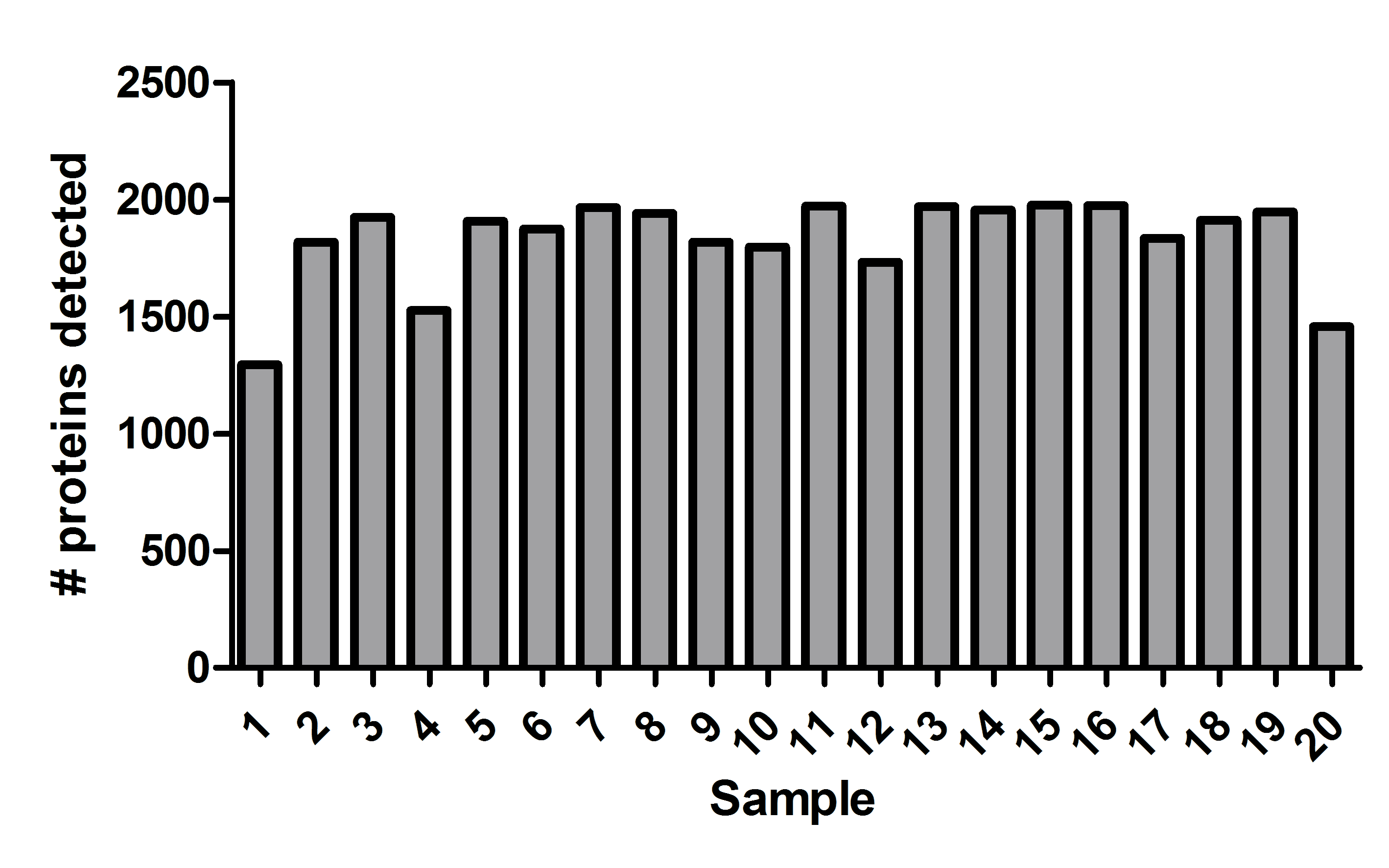

Supplement: Supplementary file 3 — Figure S3. Number of proteins detected per individual case. Proteins were quantified based on a minimum of one peptide and adhering to an FDR of < 0.01. (TIF 19662 kb) [file 40478_2018_540_MOESM3_ESM.tif]

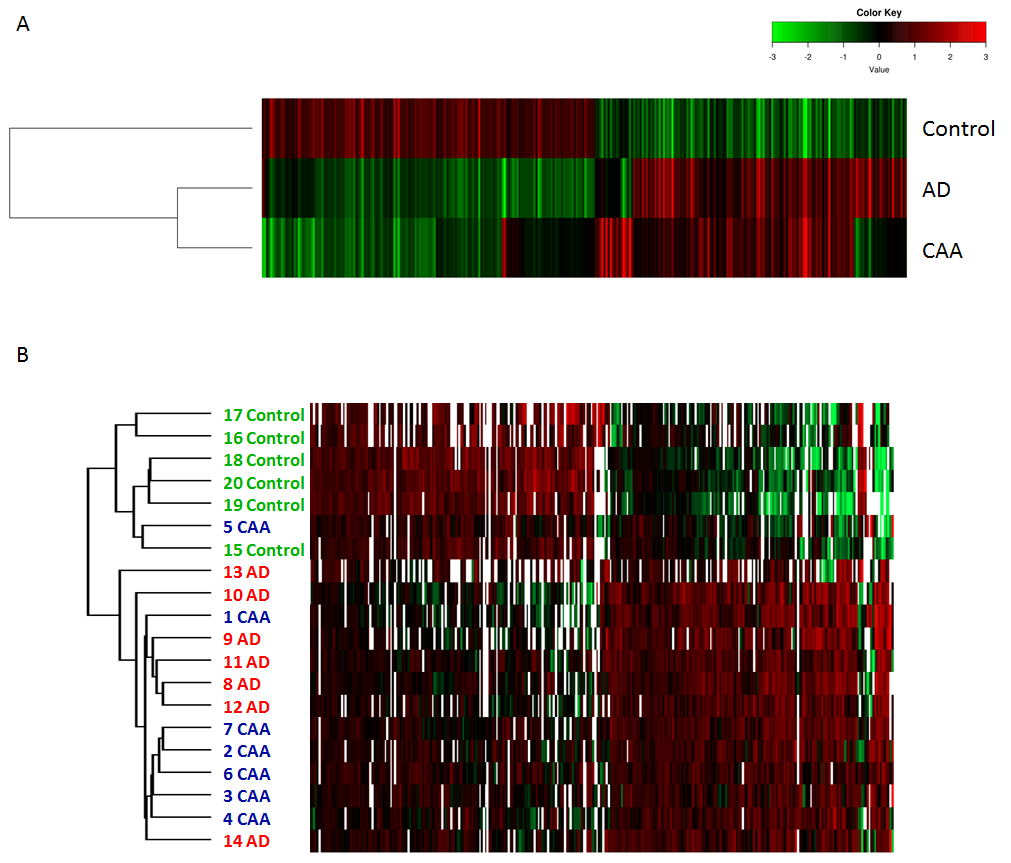

Supplement: Supplementary file 5 — Figure S4. Clustering analysis of experimental groups and individual cases. Clustering analysis and heat maps of the different experimental groups (A) and individual cases (B) based on proteins with a significant difference (ANOVA, p < 0.05) in expression between any of the groups. (TIF 709 kb) [file 40478_2018_540_MOESM5_ESM.tif]

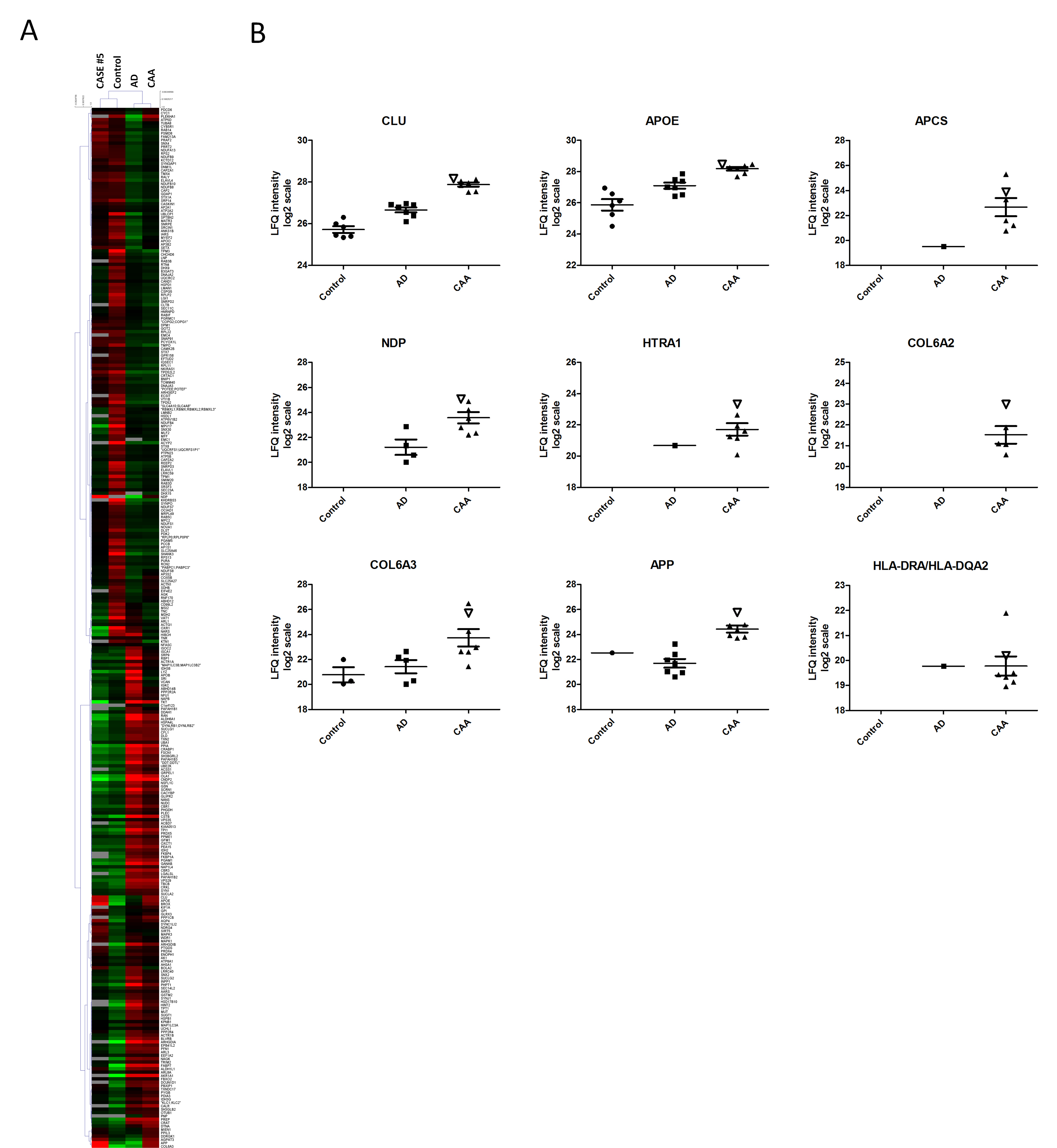

Supplement: Supplementary file 6 — Figure S5. Protein expression of CAA case #5 relative to the experimental groups and individual cases. (A) On the left the expression profile of case #5 compared to the average expression profile of the control group (2nd row), AD group (3rd row) and the CAA group (4th row). Green, expression below the overall mean; red, above the overall mean. The expression profile of case #5 is largely similar to that of the control groups but some proteins show a similar expression as in the AD and/or CAA groups. (B) Expression values (LFQ values) of several CAA specific proteins identified in this study with case #5 indicated as empty triangle pointing down. Case #5 does not differ from the CAA group in these markers. (TIF 1835 kb) [file 40478_2018_540_MOESM6_ESM.tif]

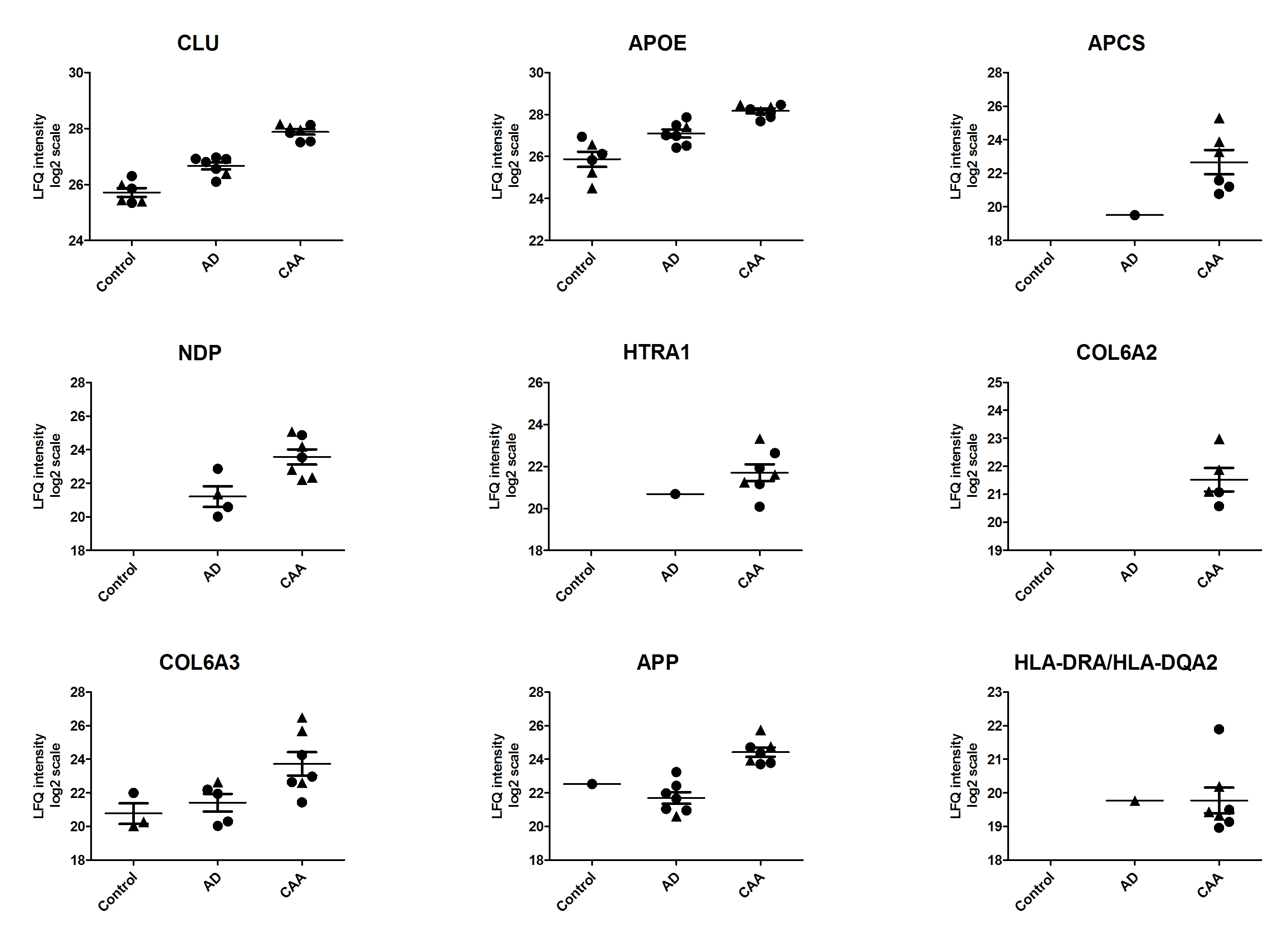

Supplement: Supplementary file 7 — Figure S6. Protein expression of males versus females. Quantitative data on several CAA selective data was plotted with males represented as triangles and females as dots. No clear relationship between gender and protein abundance was observed. (TIF 24739 kb) [file 40478_2018_540_MOESM7_ESM.tif]

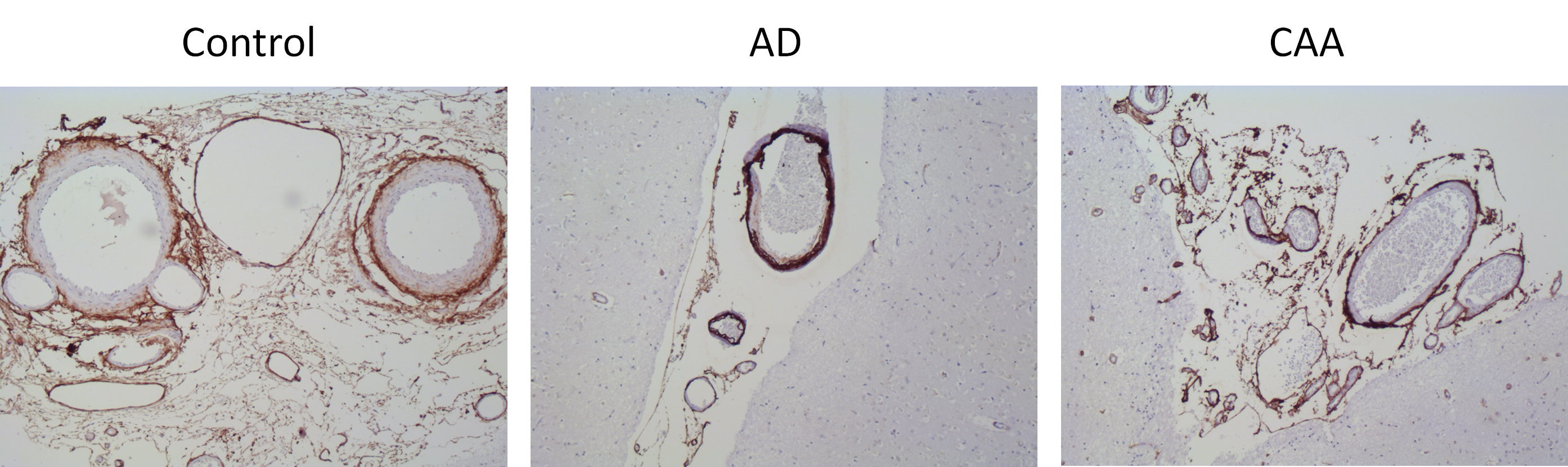

Supplement: Supplementary file 8 — Figure S7. Immunoreactivity for COL6A2 is equally present in leptomeningeal vessels in control, AD and CAA tissue. (TIF 3794 kb) [file 40478_2018_540_MOESM8_ESM.tif]

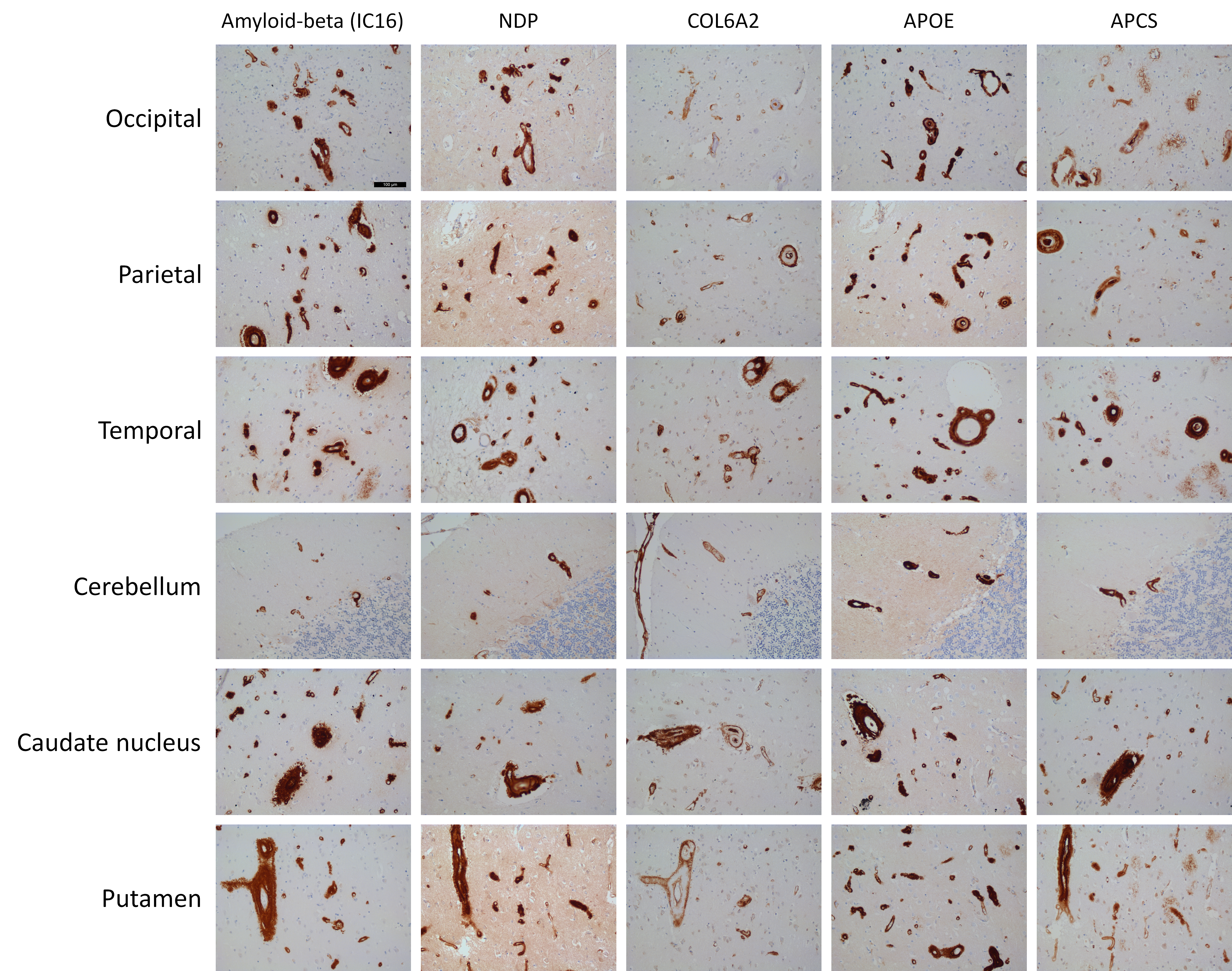

Supplement: Supplementary file 9 — Figure S8. Immunohistochemistry of Amyloid-beta, NDP, COL6A2, APOE and APCS on multiple brain regions of a HCHWA-D CAA type-1 case. Brain tissue of a case exhibiting a hereditary form of CAA type-1 was analyzed by immunohistochemistry of Amyloid-beta, NDP, COL6A2, APOE and APCS. Aβ pathology was confirmed and immunoreactivity associated with CAA type-1 pathology was found present for all markers. Scale bar in upper left picture represents 100 μm. (TIF 34988 kb) [file 40478_2018_540_MOESM9_ESM.tif]
